# An integrated analysis of cell-type specific gene expression reveals genes regulated by REVOLUTA and KANADI1 in the Arabidopsis shoot apical meristem

**DOI:** 10.1371/journal.pgen.1008661

**Published:** 2020-04-15

**Authors:** Hasthi Ram, Sudeep Sahadevan, Nittaya Gale, Monica Pia Caggiano, Xiulian Yu, Carolyn Ohno, Marcus G. Heisler

**Affiliations:** 1 European Molecular Biology Laboratory, Meyerhofstrasse, Heidelberg, Germany; 2 National Agri-Food Biotechnology Institute, SAS Nagar, Mohali, Punjab, India; 3 School of Life and Environmental Sciences, University of Sydney, NSW, Australia; National University of Singapore and Temasek Life Sciences Laboratory, SINGAPORE

## Abstract

In the *Arabidopsis thaliana* shoot apical meristem (SAM) the expression domains of Class III *Homeodomain Leucine Zipper (HD-ZIPIII)* and *KANADI (KAN)* genes are separated by a narrow boundary region from which new organs are initiated. Disruption of this boundary through either loss of function or ectopic expression of *HD-ZIPIII* and *KAN* causes ectopic or suppression of organ formation respectively, raising the question of how these transcription factors regulate organogenesis at a molecular level. In this study we develop a multi-channel FACS/RNA-seq approach to characterize global patterns of gene expression across the *HD-ZIPIII*-*KAN1* SAM boundary. We then combine FACS, RNA-seq and perturbations of *HD-ZIPIII* and *KAN* expression to identify genes that are both responsive to REV and KAN1 and normally expressed in patterns that correlate with REV and KAN1. Our data reveal that a significant number of genes responsive to REV are regulated in opposite ways depending on time after induction, with genes associated with auxin response and synthesis upregulated initially, but later repressed. We also characterize the cell type specific expression patterns of auxin responsive genes and identify a set of genes involved in organogenesis repressed by both REV and KAN1.

## Author summary

The plant hormone auxin promotes the formation of lateral organs such as leaves and flowers in a specific region of the shoot called the peripheral zone. Although the restriction of organogenesis to the peripheral zone is known to depend on the Class III *Homeodomain Leucine Zipper* (*HD-ZIPIII*) and *KANADI1* (*KAN1*) genes, the transcriptional pathways downstream of these genes have not been studied in the shoot. In this study we investigate regulatory interactions between *REVOLUTA* (*REV*), *KAN1* and auxin by developing a cell-type specific transcriptomics approach to analyse gene expression patterns and responses to perturbations. Using this approach, we identify cell-type specific genes that respond to changes in REV and KAN1 expression in the shoot. Our data reveal that while REV promotes auxin-related gene expression over the short term, both REV and KAN1 repress auxin induced genes over the long-term, consistent with their influence on organogenesis. We also identify a common set of genes repressed by REV and KAN1 that promote organogenesis.

## Introduction

The plant shoot apical meristem (SAM) is a dynamic tissue that maintains pluripotent stem cells at its center while generating lateral organs such as leaves and flowers from its periphery. Organogenesis is triggered by the local accumulation of the plant hormone auxin [1], which accumulates periodically at sites of organ formation due to a positive feedback loop between polar auxin transport and the activity of *Auxin Response Factor 5* (*ARF5*) or *MONOPTEROS* (*MP*) [2, 3]. However, the response of the meristem to auxin in terms of organogenesis and auxin transport is confined to the meristem peripheral zone. For instance, auxin application to the center of *pin1* mutant meristems only triggers organogenesis in the meristem periphery [1]. Like-wise, changes to PIN1 polarity in response to altered MP activity only occur within the peripheral zone [3]. Recent work reveals that the positioning of organs in the peripheral zone depends on the function of genes involved in leaf dorsoventrality. Genes encoding the Class III *homeodomain-leucine zipper* (*HD-ZIP III*) transcription factors are expressed centrally in the meristem while the *KANADI* (*KAN*) genes are expressed in the periphery. New organ initiation sites, marked by high levels of PIN1 expression and convergent patterns of polarity in the epidermis, are centered on cells located between these two expression domains, although high auxin levels at the initiation site subsequently promote the expansion of REV expression into the developing primordium [4]. Since loss of function mutations in either set of genes results in ectopic organ formation while ectopic expression of either type of gene can suppress new organ formation, these genes are thought to act within their expression domains to suppress organogenesis. A major challenge is to understand how this regulation is accomplished.

Previous work on seedlings has suggested that the suppression of organogenesis by these genes may occur through their direct repression of auxin transcriptional response since the Auxin Response Factors (ARFs), ARF3 and ARF4 are known to work in conjunction with KAN1 and KAN2 to repress ectopic growth from ventral leaf tissues [5]. Furthermore, ARF2, ARF3 and ARF4 are known to directly repress *WOX1* and *PRESSED FLOWER* (*PRS*) in the same leaf domain [6]. Lastly, several previous studies have shown that auxin transcriptional reporter genes appear restricted in their response to auxin in the inflorescence meristem and leaves [4, 7, 8]. So far, target gene analysis for HD-ZIPIII transcription factor REVOLUTA (REV) and KAN1 has focused on seedling tissue and on leaf development in particular. Also, although several studies support a role for the *KAN* genes in repressing auxin-related genes [9–13], the HD-ZIPIII proteins have been mostly associated with promoting auxin responses, primarily through the activation of genes involved in auxin synthesis [12, 14]. Hence it remains unclear how the *HD-ZIPIII* genes repress organogenesis and whether the genes responsive to REV and KAN1 in seedlings are similar in the shoot meristem.

In this study we investigate the role of KAN1 and REV in the shoot. The shoot meristem however is a complex structure with many distinct cell types, each expressing distinct sets of genes. Potentially each of these cell types may also respond in different ways to the same transcription factor depending on interaction partners and co-expressed regulators. To address the complexity of this tissue we utilize live-imaging combined with perturbations to REV and KAN1 expression. We also develop a combinatorial FACS-based transcriptomics approach, using several GFP variants to identify gene expression profiles specific to REV, KAN1 and boundary cell types which also alter their expression in response to REV and KAN1 perturbations. Our results reveal that although both REV and KAN1 suppress organ formation, the way in which this is achieved is different in each case. While REV expression appears capable of blocking auxin signaling in a generic manner, this is an indirect consequence of REV expression which contrasts with short term responses. In contrast, KAN1 influences gene expression more consistently but also more specifically in terms of individual auxin-regulated genes. Finally, our results also identify other hormone pathways regulated by these genes that are known to be critical for organogenesis.

## Results and discussion

### Transcriptional profiling of cells located across the REV-KAN1 boundary domain of the SAM

**Fig 1:**
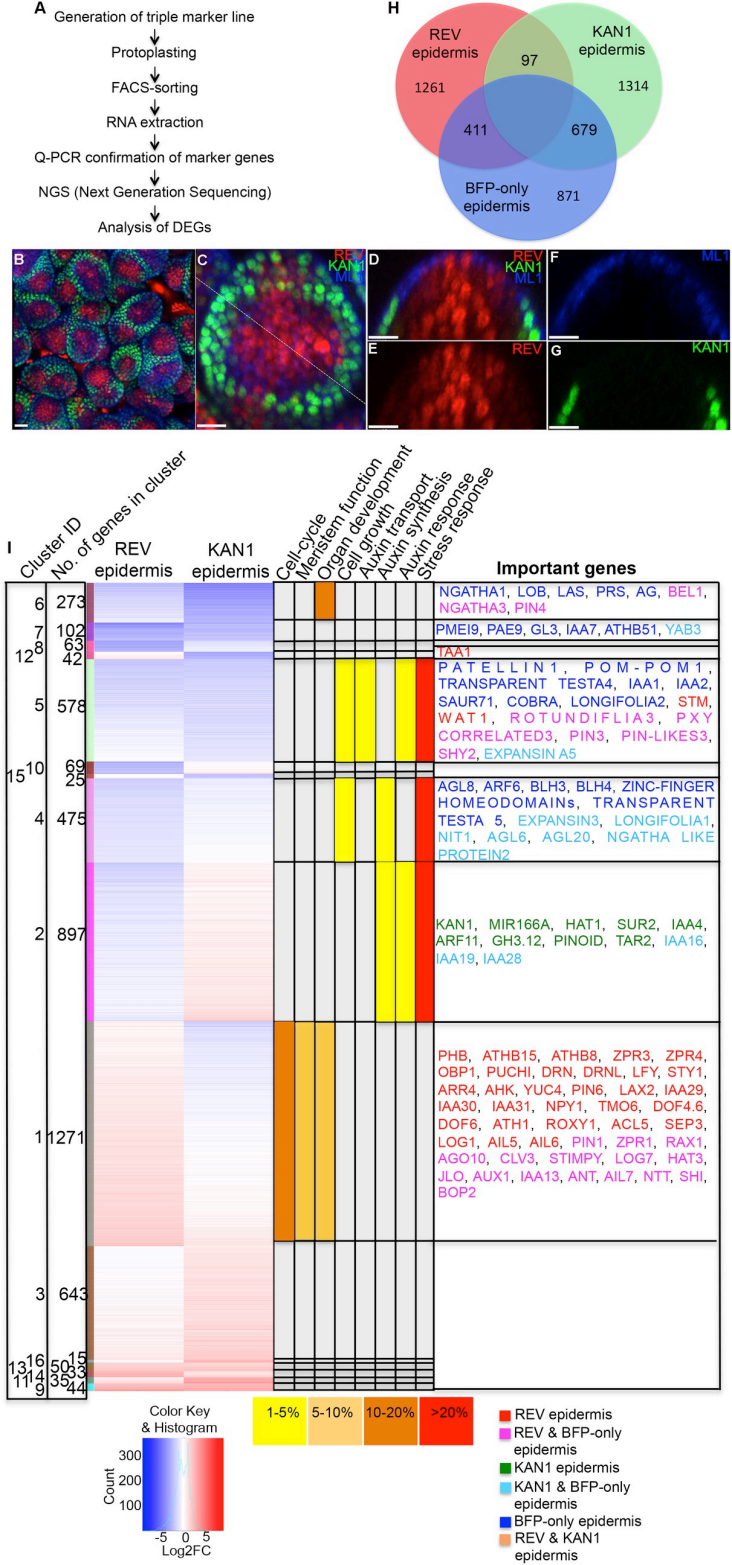
Identification of cell-type specific transcriptome. (A) Flow diagram illustrating the methodology used for cell-type specific transcriptomics. (B-G) Confocal images of *pREV*::*REV-2YPET* (Red), *pKAN1*::*KAN1-2GFP* (green) and *pAtML1*::*mTag-BFP-ER* (blue) expression in an *ap1cal* mutant SAM. (B-C) Volume rending of SAM as viewed from above showing the expression of all three fluorescent markers. (B) Zoom-out view to show multiple SAMs. (C) Zoom-in of an individual SAM. The dashed line marks the position of the longitudinal section shown in D-G. (D) Reconstructed longitudinal section of the SAM presented in C showing all three markers. (E) Same view as in D but only showing REV expression. (F) Same view as in D but only showing BFP expression. (G) Same view as in D but only showing KAN1 expression. (H) Venn diagram indicating the extent of overlap between sets of genes found to be differentially expressed in at least one of the cell types labeled by *pREV*::*REV-2YPET*, *pAtML1*::*mTag-BFP-ER* and *pKAN1*::*KAN1-2GFP*. Genes were considered as DEGs if they exhibited a log2 Fold Change (FC) >1 in any one cell-type compared with either of the other two cell-types, with an adjusted p-value <0.05. All the DEGs were classified into 6 sectors. (I) Consensus clustering of genes identified as DEGs based on common expression patterns. Cluster identification numbers are listed in the left-most column, followed by a column indicating the number of genes in each cluster. The third column indicates, with specific color bars, which genes in the adjacent expression level columns are included in each cluster. Note color intensity within the columns labelled REV and KAN1 epidermis indicates expression level for each gene in those cell types relative to expression in BFP-only cells. The color look-up-table is shown below these columns. The degree of enrichment in each cluster for selected GO terms is shown in the next several columns, with the colors indicating the percentage of genes present in the clusters associated with those GO terms. The far-right column lists some development related genes contained in the respective cluster. The cell-type enrichment patterns for these genes is indicated by the text color. Scale bars, 10 μm in B-G.

The *HD-ZIPIII* and *KAN* genes play a key role in the *A*. *thaliana* SAM by helping to specify organ positioning, cell type patterning and organ morphogenesis [4]. Identifying the global regulatory network underlying the function of these genes is therefore a major priority for advancing our understanding of these processes. We approached this challenge using a strategy that combines RNA-seq-based cell type profiling with RNA-seq-based identification of genes differentially expressed in response to transcription factor perturbations. Previous approaches for transcriptome profiling specific cell types in plants, including studies utilizing FACS, INTACT (isolation of nuclei tagged in specific cell types) and TRAP (translating ribosome affinity purification), have been limited to the collection of a single cell-type using one specific promoter at a time [15–18]. However our previous work demonstrated not only the importance of the REV and KAN1 expression domains but also specifically their epidermal expression and the importance of epidermal cells located in between their domains [4]. Taking into account these findings we developed a combinatorial FACS based transcriptomics approach that utilized three distinct fluorescent proteins to label REV, KAN1 and all epidermal cells (Fig 1A). To label all epidermal cells we used the *A*. *thaliana Meristem Layer1* (*AtML1*) promoter [19] to drive the blue fluorescent protein mTag-BFP [20] fused to an endoplasmic reticulum localization sequence (mTag-BFP-ER). To label REV and KAN1 expressing cells we used functional fluorescent protein translational fusions to these proteins under their own regulatory control (*pREV*::*REV-2YPET* and *pKAN1*::*KAN1-2GFP*) as described previously [4]. Cells only expressing mTag-BFP-ER are predicted to be epidermal cells predominantly located in the peripheral zone in between the KAN1 and REV domains [4]. The three markers were transformed consecutively into an *apetala1****-****1 cauliflower1****-****1* (*ap1cal*) double mutant plant line in order to combine them and enable the collection of large numbers of protoplasts derived from meristem tissues as described previously [21]. Imaging these transgenic lines confirmed their predicted expression patterns (Fig 1B–1G). From FACS sorting of protoplasts from these plants, six different cell-types were collected, namely epidermal REV (sorting for double positive for BFP and YFP), epidermal KAN1 (sorting for double positive for BFP and GFP), epidermis without REV or KAN1 (sorting for BFP-only), sub-epidermal REV (sorting for YFP alone), sub-epidermal KAN1 (sorting for GFP alone) and all negative (sub-epidermal cells not expressing any fluorescent protein) (S1 Fig). Q-PCR assays, using RNA extracted from the collected protoplasts, for genes known to be expressed specifically in these cell types confirmed the purity of the sorting and enrichment of marker gene expression (S2A–S2C Fig). Isolated RNAs were then sequenced using Illumina HiSeq 2000. Principle component analysis (PCA) of the sequencing data revealed close clustering of biological replicates, and separation of the cell-type profiles (S2D Fig). PCA analysis further indicated that most variation could be explained by differences in epidermal vs non-epidermal identities as well as KAN1 vs non-KAN1 expressing cells (S2D Fig). To identify genes that are differentially expressed between the cell types, we grouped the six cell types into two groups: epidermal and sub-epidermal and then compared one cell type against the remaining two cell-types within their own epidermal or sub-epidermal group. We considered a gene differentially expressed if it exhibited a log2 Fold Change (FC) of greater than 1 in at least one cell-type compared to the any of the remaining two cell-types within the group, with an adjusted p-value of <0.05. With these criteria, we identified 1769 genes up-regulated in epidermal REV cells, 2090 genes up-regulated in KAN1 epidermal cells and 1961 genes up-regulated in BFP-only cells (S1 Table). Through further analysis, we identified different subsets of genes that exhibited distinct enrichment patterns between the epidermal cell-types (Fig 1H, S2 Table). Similarly, in the sub-epidermis we identified 2330 genes up-regulated in REV expressing cells, 1211 genes up-regulated within the KAN1 domain and 2153 genes in cells expressing neither REV nor KAN1 (S3 Table). Overall our approach was successful in identifying more than 4600 genes differentially expressed in at least one of the three epidermal cell types out of around 22,000 genes with expression detected in the SAM. Comparing to previous studies, our results indicate good concordance. For instance, 186 genes were previously identified to be upregulated in KAN1 expressing cells compared to other SAM cell types (S4 Table) [18]. Of these, 76% were overlapping with our total cell-type specific genes, and of these overlapping genes, 44% were specifically upregulated in KAN1 expressing SAM cells (S3 Fig). Overall however we detected many more such genes (1314), possibly because of differences in the cell types compared as well as a higher sensitivity for RNA-seq compared to microarray analysis. To further check the accuracy of our analysis, we examined the published expression patterns of genes for which expression data are available by means of RNA *in-situ* hybridization, GUS staining or fluorescence reporters. Of 48 such genes in our analysis, 44 (92%) of them were enriched in the predicted cell-types (S5 Table). We also examined the expression of 14 genes that had either not been characterized before or not had their expression pattern examined with reference to other markers such as PIN1-GFP or REV-YFP/KAN1-GFP. Of these, 10 exhibited expression patterns that matched predicted patterns based on our FACS RNA-seq data (S4 Fig). Two of the remaining markers did not exhibit expression in the SAM suggesting that these reporters may not accurately reflect the expression pattern of the corresponding gene.

### Clustering and GO term enrichment for clusters

We next focused our expression analysis on epidermal cell-types. From here onwards, unless stated otherwise, the terms ‘REV domain/cells’ and ‘KAN1 domain/cells’ are used to denote REV epidermal and KAN1 epidermal cells, respectively while ‘BFP-only’ refers to those epidermal cells not labelled with the REV and KAN1 markers. To gain an overview of differential gene expression in the SAM epidermis, we used consensus clustering and identified 15 sets of genes with distinct patterns of expression with respect to the three cell types (Fig 1I). Gene Ontology (GO) enrichment analysis of these clusters identified significant GO terms associated with various biological functions (S6 Table). Notably, genes associated with the GO terms organ development, cell growth, auxin response and auxin transport were overrepresented in clusters 5 and 6, which represent genes that are expressed relatively highly within BFP-only cells. Genes associated with organ development as well as cell-cycle and meristem function were enriched in Cluster 1, which represents genes expressed relatively highly in the REV domain (S6 Table, Fig 1I). Genes associated with auxin synthesis, auxin response and stress response were significantly enriched in clusters 2, and 4, which represent genes expressed relatively highly in the KAN1 domain as well as BFP-only cells. We also note that genes related to stress were also enriched in clusters marked by relatively low REV expression, although this did not apply to all such clusters (Fig 1I). Example of specific genes associated with these clusters and GO terms are given in the supplementary results (S1 Text).

### Identification of REV and KAN1 responsive genes in the SAM

Having identified genes that are differentially expressed between the REV, KAN1 and BFP-only cell types, we next asked which genes alter their expression levels in response to perturbations in REV or KAN1 expression. To increase the specificity of our experiment we again focused on the epidermis by limiting our ectopic expression perturbations to the epidermis and by specifically isolating epidermal cells for RNA-seq based analysis. In two sets of experiments we either induced YFP tagged MIR165/166-resistant REV (REVr-2VENUS) or GFP tagged KAN1 specifically in the epidermis using the *AtML1* promoter and dexamethasone regulated *GR-LhG4/6XOP* two-component system [22]. We refer these two constructs as *pAtML1>>REVr-2VENUS* and *pAtML1>>KAN1-2GFP*, respectively. RNA was then purified specifically from epidermal cells using FACS, in an *ap1cal* mutant background using the ectopically expressed tagged REV and KAN1 proteins for sorting. The phenotypic consequences of these perturbations in *ap1cal* mutants were similar to those found for the wild-type, where organogenesis is suppressed and meristems become pin-shaped [4] (S5 Fig). For both the ectopic REV and KAN1 experiments we sorted the epidermal cells using FACS, 6 and 16 hours (hrs) after induction (S6 Fig). For our control experiment we collected epidermal cells labelled by BFP-ER after the same time periods of dexamethasone mediated induction of GR-LhG4, under the control of the 6XOP promoter using *pAtML1*::*GR-LhG4*, in the *ap1cal* genetic background containing *pAtML1*::*BFP-ER*. Q-PCR analysis on extracted RNA confirmed successful sorting and the regulation of known target genes of REV and KAN1 (S7A & S7B Fig). In addition, we also measured gene expression changes using whole *ap1cal* inflorescence meristems after inducing miRNA165a to knock-down the *HD-ZIPIII* genes using a two component *pAtUBQ10*::*GR-LhG4/6XOP* system (*pAtUBQ10>>miRNA165a*). For RNA-seq followed by induction of miR165a, we used whole *ap1cal* SAMs 8 or 16 hrs after induction of the transgene. As a control we induced, *pAtUBQ10*::*GR-LhG4* alone, also in the *ap1cal* background. Western blotting for REV protein and Q-PCR for REV direct target gene, *ZPR3*, revealed successful knock-down of REV in the meristem tissue (S7C & S7E Fig). Tables 1, S7, S8 and S9 report the up and down regulated genes at each time point for the RNA-Seq experiments. Known REV regulated genes such as *ZPR1*, *ZPR3*, were found to be differentially regulated by REV in both knock-down and ectopic experiments, suggesting overall good data quality. However, fewer differentially expressed genes (DEGs) were identified after miR165a induction compared with the ectopic REV/KAN1 experiment, possibly reflecting the reduced specificity of this experiment (Tables 1 & S9). We therefore focused our remaining analysis on the ectopic expression experiments.

**Table 1:** Summary of the number of DEGs identified in various RNA-Seq experiments in different transgenic lines.

Transgenic line	Time-points	Up	Down
*pAtML1>>REVr-2VENUS*	6 hrs 16 hrs	418 356	433 1370
*pAtML1>>KAN1-2GFP*	6 hrs 16 hrs	556 1227	946 2910
*pAtUBQ10>>miRNA165a*	8 hrs 16 hrs	85 183	65 250

**Fig 2:**
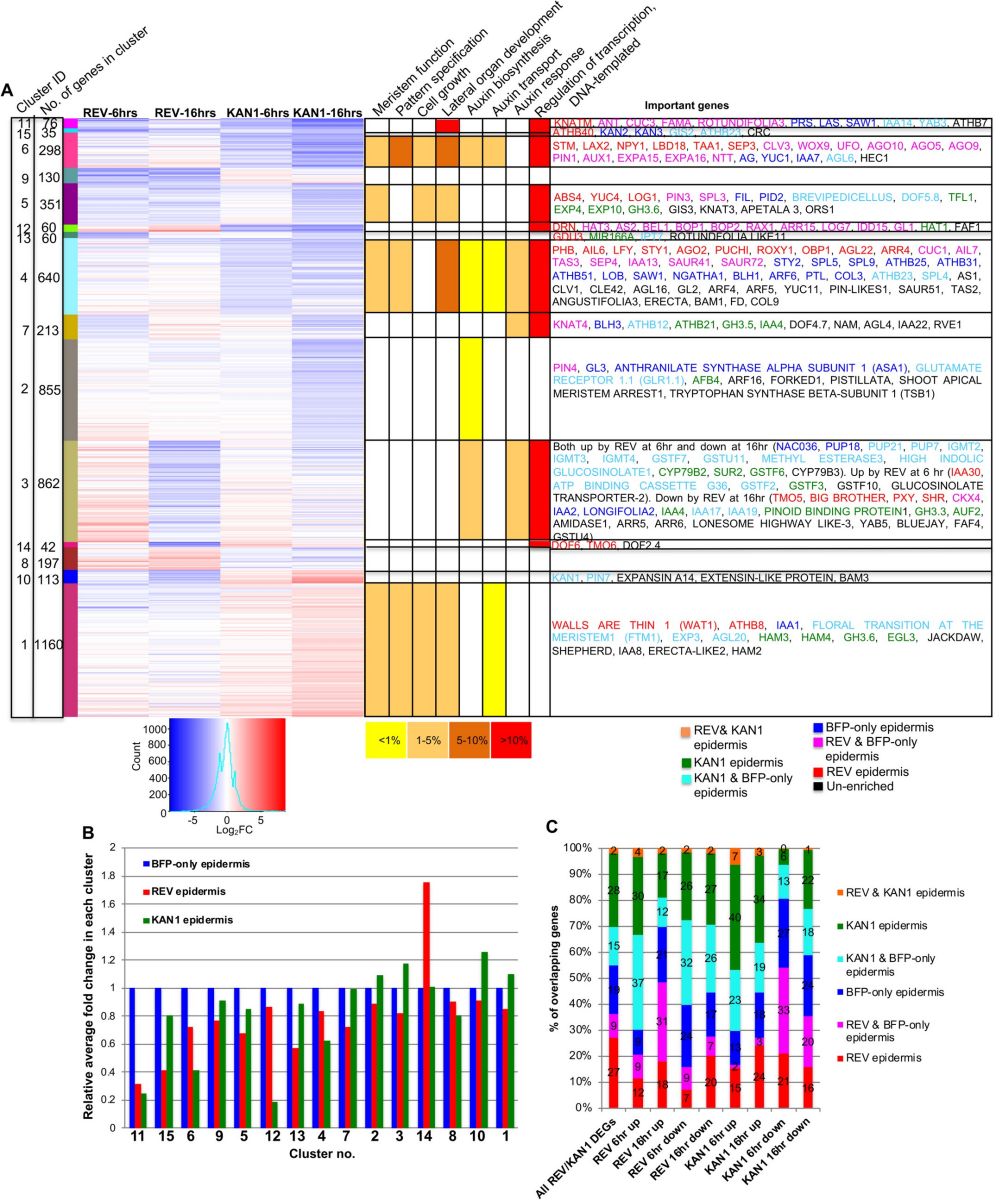
Identification of relevant REV and KAN1 responsive genes in SAM. (A) Consensus clustering of all the genes found to be differentially regulated by either REV or KAN1 at 6 hrs and 16 hrs in *pAtML1>>REVr-2VENUS* or *pAtML1>>KAN1-2GFP* transgenic lines. Cluster identification numbers are listed in the left-most column, followed by a column indicating the number of genes in each cluster. The third column indicates, with specific color bars, which genes in the adjacent expression level columns are included in each cluster. The columns that indicate fold-changes in expression are labelled according to their corresponding experiments at the top, with fold-changes in expression indicated according to the color code below these columns. To the right of the expression level columns, enrichment of selected GO terms in each cluster is indicated by color code (color code indicates the percentage of genes present in the clusters associated with the GO term). The far-right column lists some development related genes contained in the respective cluster. The cell-type enrichment patterns for these genes is indicated by the text color (the color correspondence to cell type is listed below the column). (B) Bar graph indicating average expression levels of DEGs in clusters 1–15 from B, in REV and KAN1 cell types, relative to BFP-only cells (shown with an expression level of 1). (C) Graph showing percentage overlap (numbers within the stacks of the bars) between sets of genes regulated by REV and KAN1 (horizontal axis) and the sets of genes enriched in specific cell types (marked by colors and legend adjacent to the graph). Note that overlap percentages should be compared to the first column in which percentage overlap between cell type enriched genes is shown for the set of genes that includes all REV and KAN1 DEGs. The numbers shown on stacked bars are % values rounded off to the nearest whole number.

Overall, the majority (81%) of genes that responded to ectopic REV or KAN1 expression were not co-regulated by both transcription factors (S10 Table). However of those genes that were, the largest class corresponded to genes that were co-repressed (54% of co-regulated genes at 6 hrs after induction) (S8A Fig). Consensus clustering of REV and KAN1 DEGs across the two time-points revealed that genes regulated by KAN1 undergo similar changes in expression at both early (6 hrs) and late time points (16 hrs) (Fig 2A). Furthermore, at 16hrs the fold change is more than at 6 hrs, suggesting a stable, accumulating influence of KAN1 on most of its target genes. In contrast, while some clusters of REV DEGs (cluster no. 2, 4, 5, 9) show a similar pattern of change at both time points, many genes (identified in cluster no. 3) were up-regulated at 6 hrs but down-regulated at 16 hrs, revealing both a consistent and as well as dynamically changing influence of REV on downstream gene expression (Fig 2A). Consensus clustering also identified different subsets of genes that are oppositely regulated by REV and KAN1, such as clusters 8, 9 and 10 while other clusters identify genes that are regulated by REV and KAN1 in a similar manner in at least one time point (cluster no. 1, 5, 7) (Fig 2A). GO enrichment analysis indicated “regulation of transcription, DNA-templated” as highly enriched in many clusters, indicating that in the SAM, REV and KAN1 regulate the expression of various transcription factors (Fig 2A, S11 Table), consistent with the results of previous studies on seedlings [12]. GO terms related with meristem function, pattern specification, cell growth and lateral organ development were enriched in clusters of genes repressed by either KAN1, REV or both, apart from cluster 1, which includes genes up-regulated by KAN1. Cluster 3, which includes genes up-regulated by REV at 6 hrs but down-regulated by REV at 16 hrs, was found to be enriched for genes associated with auxin biosynthesis and response. Overall there was significant overlap in the set of DEGs identified in this study with those from previous studies (S12 Table). Included in this overlap were genes belonging to gene families for which several members had been identified previously as REV or KAN1 regulated in seedlings. Such gene families include the *BEL1-like homeodomain* (*BLH*), Class II *HD-ZIP*, *INDETERMINATE DOMAIN* (*IDD*), *ZINC FINGER PROTEIN* (*ZFP*) and *FANTASTIC FOUR* (*FAF*) families of transcription factors as well as auxin-related genes including members of the *SMALL AUXIN UP RNA (SAUR)*, *PIN*, *INDOLE-3-ACETIC ACID INDUCIBLE* (*IAA)*, *LIKE AUX1* (*LAX*), *YUCCA* (*YUC*) and *NAKED PINS IN YUC MUTANTS* (*NPY*) gene families (S9 Fig). However in many cases, additional members of these gene families were found to be DEGs only in this study while other genes within these families were only identified in previous seedling-based studies (S9 Fig). Notably, members of the same gene family often appeared to be regulated similarly. For instance, all differentially expressed members of the *BLH* family were repressed by either REV, KAN1 or both while the Class II *HD-ZIP* genes identified are induced by REV and repressed by KAN1. Most members of auxin associated gene families were also repressed by REV or KAN1 or both, although a small minority were induced (S9 Fig). We also identified the hormone cytokinin to be a major focus of both REV and KAN1. For instance, the biosynthetic gene *LONELY GUY 7* (*LOG7*) (*AT5G06300*), and cytokinin responsive gene *ARABIDOPSIS RESPONSE REGULATOR 15* (*ARR15*) (*AT1G74890*) were positively regulated by REV in both knock-down and ectopic experiments (S7 & S9 Tables). In contrast, KAN1 acted mainly negatively, regulating the cytokinin biosynthesis genes [*ISOPENTENYL TRANSFERASE 7* (*IPT7*), *LOG1*, *LOG3*, *LOG7*], *HISTIDINE KINASEs* (*HK1*, *HK3*), *CYTOKININ RESPONSE FACTORs* (*CRF1*, *CRF4*, *CRF7*, *CRF10*), *ARABIDOPSIS RESPONSE REGULTORs* (*ARR3*, *ARR4*, *ARR5*, *ARR6*, *ARR15*), *CYTOKININ OXIDASEs* (*CKX1*, *CKX3*, *CKX4*, *CKX5*, *CKX7*), *KISS ME DEADLY* genes (*KMD1*, *KMD3*, *KMD4*), *PURINE PERMEASEs* (*PUP1*, *PUP4*, *PUP7*, *PUP8*, *PUP10*, *PUP14*, *PUP18*, *PUP21*) (Table 2).

**Table 2:** Regulation and expression of genes involved in cytokinin signaling.

Gene name	Gene ID	REV 6hr FC	REV 16hr FC	KAN1 6hr FC	KAN1 16hr FC	Epidermal expression domain
IPT7	AT3G23630	-2.12			-2.07	BFP-only, KAN1
LOG1	AT2G28305		-1.58		-1.27	REV
LOG3	AT2G37210	2.17			-2.13	
LOG7	AT5G06300	1.69	1.80	-2.25	-2.71	REV, BFP-only
ARR3	AT1G59940				-2.10	
ARR4	AT1G10470			-1.35	-1.37	REV
ARR5	AT3G48100		-3.07		1.15	
ARR6	AT5G62920		-2.31		-1.51	
ARR15	AT1G74890		1.66	-1.60	-2.43	REV, BFP-only
HK1	AT2G17820				-1.87	REV
HK3	AT1G27320			-1.61	-1.76	
AHP6	AT1G80100		-1.81	2.05	4.16	REV, KAN1
CRF1	AT4G11140			1.29	2.09	
CRF4	AT4G27950				-2.43	
CRF7	AT1G22985				-1.57	REV, KAN1
CRF10	AT1G68550				-1.91	
CKX1	AT2G41510				-1.17	
CKX3	AT5G56970				-1.41	KAN1
CKX4	AT4G29740		-1.38			REV, BFP-only
CKX5	AT1G75450				-1.29	
CKX7	AT5G21482				-1.04	
KMD1	AT1G80440	-1.87		-1.35	-3.92	BFP-only, KAN1
KMD3	AT2G44130				-2.05	
KMD4	AT3G59940				-1.78	
PUP1	AT1G28230	-3.18	-3.83		-2.69	BFP-only, KAN1
PUP4	AT1G30840		-1.97	1.65	3.35	KAN1
PUP7	AT4G18197	2.65	-1.03			BFP-only, KAN1
PUP8	AT4G18195					BFP-only, KAN1
PUP10	AT4G18210			-1.37	-2.01	BFP-only, KAN1
PUP14	AT1G19770					BFP-only, KAN1
PUP21	AT4G18205	3.20	-1.28			BFP-only, KAN1
PUP18	AT1G57990	1.45	-1.12		-1.05	BFP-only

### Complementary transcriptomics approach identifies cell-type specific genes responsive to REV and KAN1

Having identified genes differentially expressed in the SAM REV, KAN1 and BFP-only epidermal expression domains as well as genes differentially expressed in response to perturbations of REV and KAN1 expression in the SAM epidermis, we proceeded to analyse the overlap between the two data sets. Firstly, we investigated whether any of the sets of genes clustered by their response to REV and KAN1 perturbations, were preferentially expressed in specific cell types. We found this to be the case for several clusters including clusters 11, 15, 6, 12, 13, 4, 14, based on a 1.5-fold cut off. For instance, cluster 11, which represents genes repressed by both REV and KAN1 were on average more highly expressed in BFP-only cells where REV and KAN1 expression is absent (Fig 2B). This cluster is enriched for genes involved in organ formation such as *AINTEGUMENTA* (*ANT*), *PRESSED FLOWER* (*PRS*), *SAWTOOTH 1* (*SAW1*) and *ROTUNDIFOLIA 3* (*ROT3*), consistent with the finding that ectopic expression of REV and KAN1 represses organogenesis [4]. Genes in clusters 6 and 12 also exhibited reduced expression in the KAN1 domain while cluster 15 shows reduced expression in the REV domain. Correspondingly, clusters 6 and 12 represent genes repressed by KAN1 while cluster 15 represents genes repressed by REV (Fig 2A). The average expression level of genes in Cluster 10 was slightly higher in KAN1 expressing cells in comparison to the other cell types and consistent with this, this cluster represents genes up-regulated by KAN1. Genes in Cluster 4 are repressed by KAN1 (Fig 2A) and consistent with this, the average expression level of Cluster 4 genes is lower in the KAN1 domain compared to BFP-only domain (Fig 2B). Perhaps surprisingly, genes in Cluster 14, which represents genes repressed by REV at 16 hrs also represents genes that are more highly expressed in the REV domain. Genes in this cluster include *DNA BINDING WITH ONE FINGER* (*DOF*) *5*.*3* (also called *TARGET OF MONOPTEROS 6*), *DOF6* and *DOF2*.*4*, which are genes also known to be expressed in vasculature [23]. Since long-term ectopic expression of REV represses organogenesis, the repression of these vascular expressed genes is most likely an indirect consequence of this phenotype.

In addition to comparing the average expression levels of specific clusters in different cell types, we also investigated the cell type specific gene expression patterns of genes responsive to REV or KAN1 (Fig 2C). Overall, we found that the majority of genes regulated by REV or KAN1 within 6 hrs, show a cell type specific expression pattern although this was true for a lower percentage of genes regulated at 16 hrs after induction (S13 Table). As might be expected, the set of genes repressed by REV at 6 hrs is made up of a large proportion (82%) of genes that are usually expressed at lower levels in the REV domain. This compares to a lower proportion (62%), if considering the set of all genes found to be differentially expressed in response to REV/KAN1 perturbations (1^st^ bar in Fig 2C). Similarly, around 80% of genes repressed by KAN1 at 6 hrs are differentially expressed at higher levels outside the KAN1 domain compared to 55% for all REV/KAN1 DEGs. For genes up-regulated by KAN1 at 6 hrs, around 70% are expressed at higher levels in the KAN1 domain (compared to 45% for all REV/KAN1 DEGs) (Fig 2C). However, for genes induced by REV at 6 hrs, 75% were found to be differentially expressed at higher levels outside the REV domain (compared to 64% for all REV/KAN1 DEGs). This percentage dropped to around 50% for genes induced by REV after the longer interval of 16 hrs (Fig 2C). We note that this finding is consistent with the characteristics of genes in Cluster 3 (i.e. Cluster 3, Fig 2A), which are initially upregulated by ectopic REV expression, but then subsequently repressed and under normal conditions more highly expressed in the KAN1 and BFP-only domain compared to the REV domain (Fig 2B). Analysis of the overlap reveals 304 out of the 862 genes in cluster 3 are differentially expressed at higher levels outside the REV domain compared to 106 of them that are expressed more highly within it. Genes related to glucosinolate metabolic processes and defense response were found to be enriched in this set of 304 genes (Fig 2A and S14 Table). Glucosinolates play an important role in defense against pathogens and Indole-glucosinolates (IGs) and Indole-Acetic Acids (IAAs) are synthesized from the common precursor tryptophan. It has also been shown that changes in IG levels in plant cells affects IAA concentrations [24, 25], suggesting the possibility that these genes may be transiently and indirectly up-regulated by REV to maintain auxin homeostasis. To check this possibility, we selected thirteen genes from this cluster and analysed their expression levels by qPCR after induction of *p35S*::*GR-REVd* in the presence and absence of the protein synthesis inhibitor cycloheximide. As shown in S10 Fig, although most of the genes were confirmed to be induced by REV, in the presence of cycloheximide treatment most genes remained uninduced confirming indirect regulation.

#### Cell type specific genes induced 16hr after REV induction are predominantly expressed in the REV and BFP-only domain

Of the genes which are induced 16 hr after induction of REV, many are expressed at higher levels in both REV and BFP-only cells (Fig 2C). Furthermore, from this overlapping gene set, the majority (47 out of 58) are also repressed by KAN1 (S15 Table). This set includes known REV targets such as the Class II *HD-ZIP* genes (*HAT3*, *HAT9*, *HAT22*) and *LITTLE ZIPPERs* (*ZPR1*, *ZPR3*, *ZPR4*) [14, 26] and known KAN1 targets such as *ASYMMETRIC LEAVES 2* (*AS2)* [27], as well as genes involved in meristem maintenance (*SEPALLATA 4*/*AGAMOUS-LIKE 3*, *CAULIFLOWER*/*AGAMOUS-LIKE 10*, *WUSCHEL*, *BELL1*, *HOMEOBOX GENE 1*(*ATH1*), *DORNROSCHEN*, *LOG7*, *ARR15*), axillary meristem initiation (*REGULATOR OF AXILLARY MERISTEMS 1* (*RAX1*)), establishment dorsal cell identity (*AS2*, *ZFP8*, *GLABRA 1* (*GL1*), *TRANS-ACTING SIRNA 3* (*TAS3*), *BLADE ON PETIOLE 1* (*BOP1*), *BOP2*) and promotion of auxin biosynthesis and transport (*IDD15*, *ANT-LIKE 5*). Auxin responsive genes in this group that also promote organ development include *DORNROSCHEN*, *NPY1* and *SHI-RELATED SEQUENCE 7* while other early auxin-responsive genes include *IAA30* and *SAUR41*, which remain to be characterized (Table 3, S16 Table).

**Table 3:** Expression dynamics of selected development related genes.

.	REV epidermis (1261)	REV & BFP-only epidermis (411)	BFP-only epidermis (871)	BFP-only & KAN1 epidermis (679)	KAN1 epidermis (1314)	REV & KAN1 epidermis (97)
**REV up 6 hr & 16 hr**	ZPR3, ZPR4, AIL5, HAT22, WAT1	TAS3, LOG7, RAX1, HAT3, AS2, BELL1, PAR2	NGATHA-LIKE PROTEIN 3		HAT1, HAN	
**REV up 6hr**	REV, IAA30, WUS, OBP1			IGMT2, IGMT3, IGMT4, FTM1	SUR2, CYP79B2	
**REV up 16hr**	AGL10, ATH1, DRN, NPY1, SHI-RELATED SEQUENCE 7,	ZPR1, GL1, BOP1, BOP2, ARR15, HAT9, SEP4, IDD15, ZFP8, SAUR41	ARGOS-LIKE, UNICORN, HAT14	ATHB13	IDD16	
**REV down 6 hr & 16 hr**	AIL6, ROXY1	ANT	KAN2, KAN3, FIL	KAN1, YAB3, KNAT1, PIN7, ATHB5, ATHB23, SAUR42, SAUR79, UNE10	EGL3, GH3.6, GH3.12	
**REV down 6 hr**	YUC4, IAA29, PHB,	CUC1, CUC3, ROTUNDIFOLIA 3	PMEI9, LAS, ATHB31, BEL1-like homeodomain 3,	YAB2, GH3.17, IPT7, KISS ME DEADLY 1, ATHB12, BEL1-like homeodomain 5	MIR166A, GH3.5, IAA4	BRANCHED 1
**REV down 16 hr**	ACL5, TMO6, DOF6, PIN6, ATHB8, ATHB40, LOG1, STY1, PXY, SHR, BIG BROTHER, REPRESSOR OF WUS 1	PIN3, AIL7, AGO5, CKX4	CLE41, PID2, PRS, IAA1, IAA2, LONGIFOLIA2	IAA14, IAA17, IAA19, DOF5.8, BANQUO 2, IGMT2, IGMT3, IGMT4	GH3.2, GH3.3, CYP79B2, SUR2, ROTUNDIFOLIA LIKE 8, PINOID-BINDING PROTEIN 1, HAM3, AUF2	BRI1-LIKE 3, AHP6, BREVIS RADIX-LIKE 1
**KAN1 up 6 hr & 16 hr**			IAA1	PIN7	GH3.2	AHP6
**KAN1 up 6 hr**	TMO6, DOF6, ATHB8,		CLE9		SAUR5, SAUR69, ROTUNDIFOLIA LIKE 8	
**KAN1 up 16 hr**	IAA29, BRI1 LIKE		HAT14, YUC3,	AGL20, FLORAL TRANSITION AT THE MERISTEM 1	HAM3, EGL3, GH3.3, GH3.4	
**KAN1 down 6 hr & 16 hr**	DRN, TERMINAL EAR-1 LIKE 2, TAA1, NPY1, ROXY1, LAX2, STM, IAA31, AS2-LIKE 39, AIL6, HAT22, STY1, LFY, IDD14, ARR4, OBP1, AGO2, TAPETUM DETERMINANT1,	BOP1, BOP2, AS2, TERMINAL EAR-1 LIKE 1, BELL1, ANT, CLV3, AGO7, PIN1, CUC3, STIMPY, NTT, HAT3, ATHB21, UFO, JLO, GL1, LOG7, AUX1, LAX1, RAX1, AGO10, ROTUNDIFOLIA 3, IAA13, IAA26, ARR15, PIN-LIKES 3, SAUR 41, AIL7, AGO9,	KAN2, KAN3, YAB1, PRS, YUC1, LAS, PTL, ARGOS-LIKE, PMEI9, ATHB25, IAA7, SAW2, STY2, AGAMOUS, NGATHA-LIKE PROTEIN 3, BEL1-like homeodomain 1	YAB2, YAB3, IAA14, BEL1-like homeodomain 5, KNAT1, AGL6, KISS ME DEADLY1		
**KAN1 6 hr down**	PUCHI, DRNL, ZPR3, ATH1, PHB	CLE21, CLE16, CLE27, TAS3, PAR1, CUC1, SAUR72, SHI	SAW1, LOB, ARF6, NGATHA 1, ATHB51, HAT14, ATHB31,	GLABROUS INFLORESCENCE STEMS 2	HAN	
**KAN1 16 hr down**	SEP3, AAO1, AGL51, AHK1, TMO6, LOG1, AIL5	NGATHA 3, CLE43, HAT9, PIN4, KNAT4,	IAA2, NO APICAL MERISTEM, GL3, LONGIFOLIA 2, BEL1-like homeodomain 3, UNICORN	ATHB12, IPT7, SAUR42, IGMT1, UNE10, ATHB23, NGATHA-LIKE 1	HAT1, TERMINAL FLOWER 1, GH3.12, ATHB21, CKX3, MIR166A, SUR2, AFB4	CYTOKININ RESPONSE FACTOR 7
**Unregulated genes**	PHV, ATHB15, AP1, BARELY ANY MERISTEM 2, HISTONES, CYCLINS, CDKs, KINESINs, INCURVATA2, YUC6, ARR9,	AS2 LIKE-1, CLV2, ATHB18, LOG4, ARR16, SHY2/IAA3,	CUC2, NIT4,	IAA16, IAA28, NIT1, NIT3, WOX12	CYPs, GSTs, KNAT2, PID	

#### Genes down-regulated by REV and excluded from the REV domain correspond to genes responsive to auxin and genes that promote ventral identity

Genes down-regulated by REV and differentially expressed at higher levels outside the REV domain include many genes involved in specifying ventral cell identity, such as *KAN1*, *KAN2*, *KAN3*, *YABBY1* [*FILAMETOUS FLOWER* (*FIL*)], *YABBY2*, *YABBY3*, *miRNA166A* (Table 3, S16 Table). While negative regulation of the *KAN* genes and *miRNA165/166* by REV has been reported previously [28], the finding that *FIL* is also repressed by REV is consistent with previous data showing that the expression of a fluorescent reporter for *FIL* expression becomes complementary to REV domain during leaf initiation [4] and that *FIL* is not expressed in the dorsalized leaves of *phb-1d* [29]. Additionally, this group of genes contains many auxin-responsive gene families such as 6 *IAA* genes (*IAA1*, *IAA2*, *IAA4*, *IAA14*, *IAA17*, *IAA19*), 4 class I *HD-ZIPs* (*ATHB5*, *ATHB12*, *ATHB23*, *ATHB31*), 6 *GRETCHEN HAGEN 3* (*GH3*) genes (*GH3*.*2*, *GH3*.*3*, *GH3*.*5*, *GH3*.*6*, *GH3*.*12*, *GH3*.*17*), 2 *SAUR* genes (*SUAR42*, *SAUR79*), *BELL* family genes (*BLH3*, *BLH5*) and genes involved in auxin transport (*PIN3*, *PIN7*, *PINOID2*, *PINOD BINDING PROTEIN 1*) (Table 3, S16 Table). These results support the recently proposed role of REV in repression of auxin signaling [4].

#### Genes up-regulated by KAN1 in the KAN1 expression domain

This group includes three *GH3* genes (*GH3*.*2*, *GH3*.*3*, *GH3*.*4*), *SAUR5*, *SAUR69* and *PIN7* (Table 3, S16 Table). *GH3* proteins function as IAA-amido synthases that conjugate Ala, Asp, Phe, and Trp to auxin. Mutant plants carrying insertions in these genes are hypersensitive to auxin, suggesting that these genes work as negative regulators of auxin abundance [30].

#### Genes down-regulated by KAN1 and excluded from KAN1 domain

As mentioned above, many genes in this category are also up-regulated by REV. Evidence that KAN1 represses these genes independently of REV comes from the finding that REV itself was not found to be repressed by KAN1 within the 16 hrs time period. However by 6 hrs we detect down-regulation of *PHABULOSA* (*PHB*) (S8 Table) indicating that the repression of these genes by KAN1 may still occur indirectly via the repression of HD-ZIPIII genes. The genes not regulated by REV but repressed by KAN1 can be divided into two groups based on their expression pattern: The first group is enriched for genes expressed in REV cells and included in this group are many genes involved in meristem maintenance and organogenesis, such as *CLAVATA3* (*CLV3*), *CLV3 RELATED* genes (*CLE16*, *CLE43*), *WOX9*/*STIMPY*, Class II *KNOTTED1-LIKE HOMEOBOX* genes (*KNAT4*), *ARGONAUTE* (*AGO*) genes (*AGO7*, *AGO9*, *AGO10*), *LATERAL ORGAN FUSION 1* (*LOF1*), *JAGGED LATERAL ORGANS* (*JLO*) (Table 3) [31–37], consistent with a repressive for KAN1 in meristem development. Other genes in this subcategory include genes involved in floral organ specification and growth (*UNUSUAL FLORAL ORGANS* (*UFO*), *NGATHA3*, *SHORT INTERNODE* (*SHI*) *NO TRANSMITTING TRACT* (*NTT*)) [38–42], auxin transport (*PIN1*, *PIN4*, *PIN-LIKES3*, *AUX1*, *LAX1*, *phospholipase D P2* [43–46], genes involved in lateral organ development and patterning such as *AGAMOUS*, *GL3*, *NGATHA1*, *PETAL LOSS*, *STYLISH 2*, *SAW1*/*BLH2*, *SAW2*/*BLH4*, *BLH1*, *LOB* [47–53] and genes involved in auxin synthesis and signaling (*YUC1*, *IAA7*, *AUXIN RESPONSE FACTOR 6* (*ARF6*)) [54–56] (Table 3, S16 Table).

#### Cell type expression analysis of co-repressed targets

In total, 2275 genes in *pAtML1>>REVr-2VENUS* and 4699 genes in *pAtML1>>KAN1-2GFP* line were found to be differentially expressed (S7 & S8 Tables) and of these, 1090 genes were common between them (S10 Table). At 6 hrs after induction, 54% of these common targets were co-repressed compared to 35% oppositely regulated while at 16 hrs, both co-repressed and oppositely regulated genes are equally represented (47% each) (S8A & S8B Fig, S17 & S18 Tables). Of the 492 genes that are co-repressed at either one of the two time points, (S10 Table), 199 (40%) are expressed more highly in BFP-only cells. Of these, 86 were found to be expressed exclusively in BFP-only cells, 35 relatively highly in both REV and BFP-only cells, and 78 genes were expressed relatively highly in both KAN1 and BFP-only cells (S10 Table). Co-repressed genes expressed exclusively in the BFP-only domain include genes involved in lateral organ outgrowth including *FIL*, *PRS*, *LONGIFOLIA 2* and *LATERAL SUPPRESSOR* (*LAS*), (S19 Table) [57–59]. Other genes in this category include genes involved in cell-wall modifications, such as *PEROXIDASE CA*, *EXTENSIN 4*, *PECTIN ACETYLESTERASE 9* and *PECTIN METHYLESTERASE INHIBITOR 9*. Genes enriched in both BFP-only and REV cells include genes involved in organ initiation such as *ANT* and *ANT-LIKE 7*, meristem organ boundary specification, such as *CUP SHAPED COTYLEDON* (*CUC*) *1* and *CUC3* and hormone degradation including *GIBBERELLIN 3-OXIDASE 3*, *CYTOKININ OXIDASE 4* and *BRASSINOSTEROID-6-OXIDASE 2*) (S19 Table). Genes enriched in both BFP-only and KAN1 cells include *YABBY2*, *YABBY3*, *KNAT1* a class I *KNOX* gene and its target involved in cytokinin biosynthesis *IPT7*, a negative regulator of cytokinin signaling (*KISS ME DEADLY 1*), known auxin responsive genes (*IAA14*, *SAUR42*) and Class I *HD-ZIP* transcription factors *ATHB12* and *ATHB23* (S19 Table).

**Fig 3:**
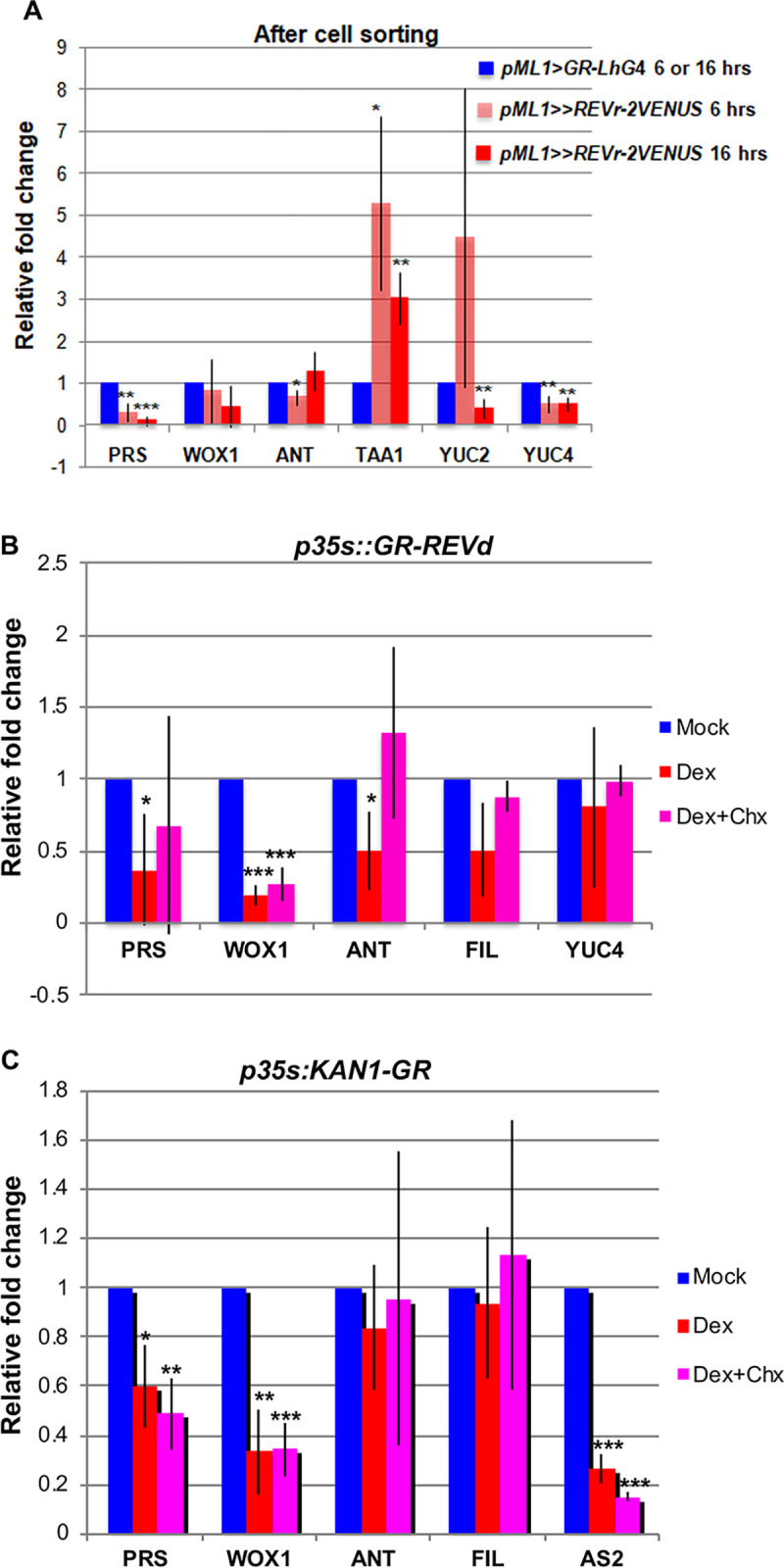
Q-PCR validation of selected genes. (A) Q-PCR analysis of selected genes in *pAtML1>>REVr-2VENUS* and *pAtML1*::*GR-LhG4;pAtML1*::*mTag-BFP-ER* lines after 6 or 16 hrs of dex treatment and cell sorting with FACS. (B) Q-PCR analysis of selected genes in *p35s*::*GR-REVd* line after mock or dexamethasone (dex) or dex + cycloheximide (chx) treatment for 3 hrs. (C) Q-PCR analysis of selected genes in *p35s*::*KAN1-GR* line after mock or dex or dex+chx treatment for 3 hrs. As dex was dissolved in ethanol, so in both B and C, mock refers to treatment with ethanol or ethanol+chx for dex and dex+chx, respectively. The values in A-C are normalized to *ACT2*. N = 3, * = p<0.05, ** = p<0.01, *** = p<0.001.

To validate our results, we measured the expression of some of the identified REV/KAN1 targets using Q-PCR in plants expressing *p35S*::*GR-REVd* and *p35S*::*KAN1-GR*, separately after dex induction (Fig 3). Q-PCR confirmed that REV and KAN1 repress the middle domain genes *WUSCHEL RELATED HOMEOBOX* (*WOX)1* and *PRS*/*WOX3* (Fig 3B and 3C). Cyclohexamide treatment did not alter this result revealing that KAN1 directly represses both of these genes, whereas REV directly represses *WOX1*, and indirectly represses *PRS* (Fig 3B and 3C). In another independent Q-PCR, in which we sorted cells after induction of *pAtML1>>REVr-2VENUS*, *PRS* expression was repressed by REV but no effect was observed on *WOX*1 expression (Fig 3A), which is consistent with the observation that *WOX1* is not expressed in the SAM [4]. Q-PCR experiments also confirmed indirect repression of the primordial genes *ANT* and *FIL* by REV, although the effect on *FIL* expression was statistically less significant (p<0.1). No effect of KAN1 on *FIL* or *ANT* was detected by Q-PCR.

All together our results identify many prominent genes known to play a role in organ development as responsive to REV/KAN1 that were not identified as such previously. Furthermore, in many of these cases, several related family members were also identified. For instance multiple members of the *BOP*, *KNAT*, *BARELY ANY MERISTEM* (*BAM*), *CUC*, *ANT-like*, *YABBY*, *SEPALLATA*, *ARGONAUTE*, *MADS*, *GLABRA*, *NGATHA*, *DORNROSCHEN (DRN)*, *RAX*, *TRANS-ACTING siRNA* and *STYLISH* gene families were identified as regulated by REV or KAN1. The vast majority of these genes were found to be repressed by KAN1 at either 6 hrs or 16 hrs after induction but many of them are also repressed by REV at the later 16 hr time point (S16 Table), consistent with the repression of organogenesis caused by ectopic expression of either REV or KAN1 [4]. We also noticed extensive regulation of cytokinin signalling related genes by REV and KAN1, some of which are expressed in domains resembling those of REV and KAN1. For instance, our data indicate that KAN1 promotes *PUP4* expression and that *PUP4* is expressed in KAN1 domain in the shoot. Other *PUP* genes are expressed in a similar pattern (S16 Table), including *PUP14* which acts to repress cytokinin signalling by depleting cytokinin in the apoplast [60]. Thus, we would expect cytokinin signalling to be repressed in the peripheral KAN1 meristem domain. In contrast, REV likely promotes cytokinin synthesis by upregulating *LOG3* and *LOG7* expression suggesting antagonistic regulation of meristem function by these genes through opposing roles in cytokinin signalling. In addition to identifying characterised genes, we also found a large proportion (27–42%) of REV/KAN1 DEGs that remain to be characterised. Some of the more predominant gene families in this class based on database annotation encode GDSL-motif esterases, major facilitator superfamily proteins, homeodomain containing proteins and zinc finger containing proteins.

### Transcriptome analysis of auxin induced organogenesis in the shoot

**Fig 4:**
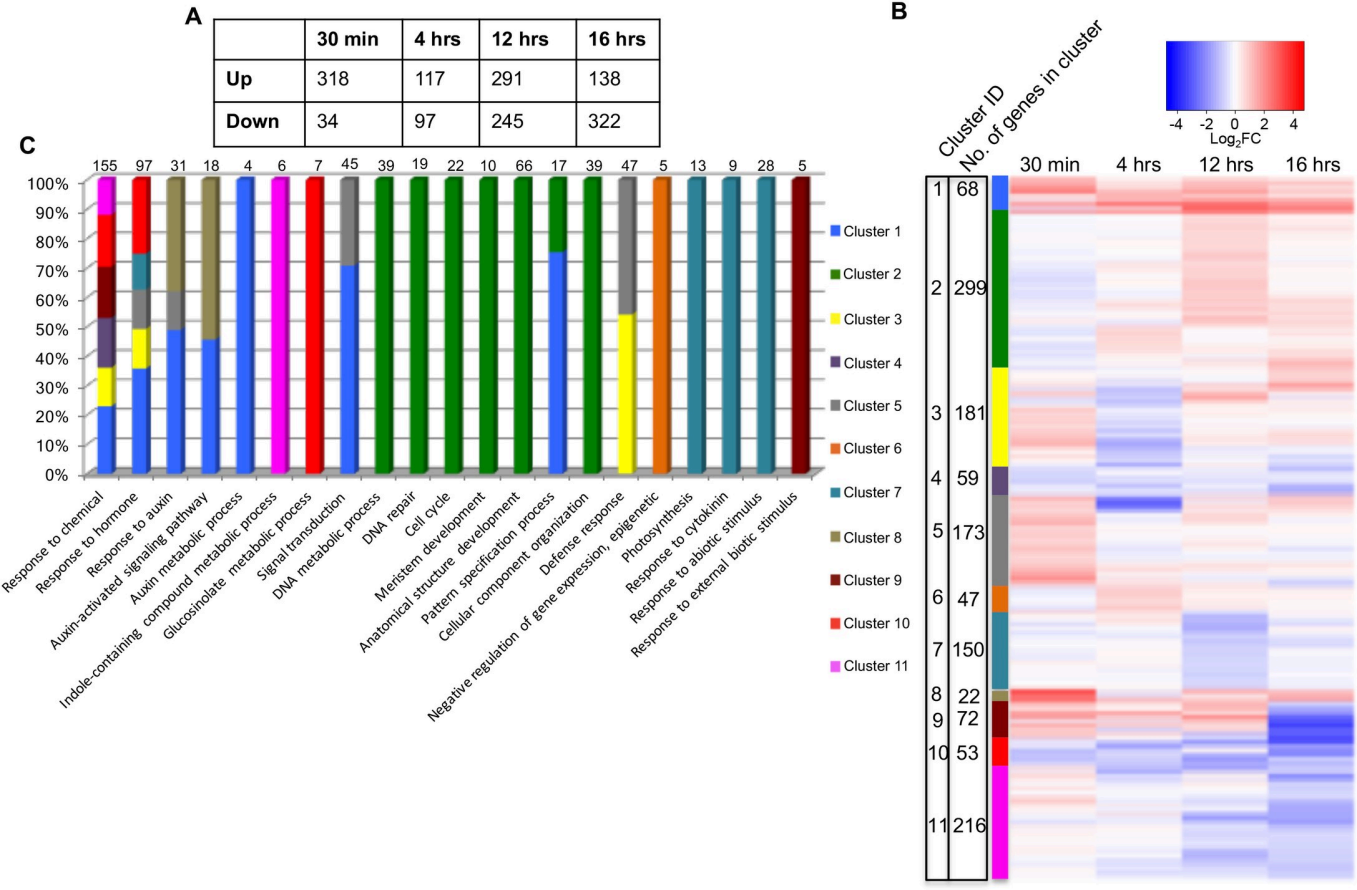
Identification of auxin-regulated genes in the Arabidopsis inflorescence meristem. (A) Summary of number of up or down regulated genes at various time points after auxin application to the *pin1* SAM. (B) Hierarchical clustering of all genes differentially expressed in response to auxin. The genes clustered according to expression were divided into 11 manually selected clusters indicated by the cluster ID and color markings adjacent to the expression level columns. (C) Representation, as a percentage, of selected Gene Ontology (GO) terms amongst the clusters identified in Fig 5B. E.g. GO term “Defense response” is represented only in clusters 3 and 5, with each cluster containing approximately 50% of genes associated with this GO term. The total number of genes associated with each GO term is marked above each GO term column.

Previous findings indicate both the HD-ZIPIII and KAN repress the formation of new lateral organs. Since organ formation is dependent on auxin-induced transcription [3, 61, 62], these observations suggest that HD-ZIPIII and KAN activity may repress auxin-induced transcription [4]. Further data supporting this proposal comes from findings that ARF3 and ARF4 work in conjunction with KAN1 and KAN2 to repress the formation of ectopic outgrowths from leaves [5]. To test this proposal further, we decided to investigate the regulatory relationship between KAN1, REV and auxin-induced gene expression in the SAM. We first identified genes involved in auxin-mediated organogenesis by applying auxin paste to *pin1* mutant meristems, which promotes organ outgrowth from the SAM periphery [1]. We then performed RNA-Seq using RNA extracted from *pin1* mutant meristems 30 min, 4hr, 12hr and 16hr after auxin application. As shown in Fig 4A, at 30 min after auxin application, most DEGs are up-regulated (318 up against 34 down), indicating that auxin mainly acts as a transcriptional activator. However at later time points, the proportion of up-regulated genes decreases and at 16 hr after auxin application, down-regulated genes outnumber up-regulated genes (Fig 4A). Of the 318 early auxin induced genes identified at 30 min after auxin application, 53 genes exhibited a log2 FC >2 and 36 of these have been previously characterized according to the TAIR database (www.arabidopsis.org) (S20 Table). Additionally we compared these 318 genes with publicly available microarray data [63–65] obtained after IAA application on seedling tissue and we found around 20% overlap (S21 Table). To identify sets of similarly regulated genes we used hierarchical clustering (Fig 4B and S22 Table). GO enrichment analysis of these clusters identified biological processes affected by our auxin treatment (Fig 4C and S22 Table). Two clusters (cluster no. 1 and 8), which represent genes up-regulated at all time points, show enrichment of the GO terms response to auxin, auxin activated signaling pathway, and auxin metabolic processes, indicating that these biological processes remain active over the term of the time course. Cluster 2, which contains genes weakly down-regulated at 30 min and up-regulated at later time points, shows enrichment of GO terms DNA metabolic process, DNA repair, cell cycle, cellular component organization, meristem development, anatomical structure development and pattern specification process, consistent with expected growth and patterning in response to the auxin treatment. Genes repressed at all time points show enrichment of the GO terms response to chemical (cluster no. 4), photosynthesis, response to cytokinin and response to abiotic stimulus (cluster no. 7). Genes strongly repressed at 12 and 16 hrs in cluster no. 10 and 11 are enriched for genes associated with the GO terms glucosinolate metabolic process and indole-containing compound metabolic process, respectively.

### The expression of auxin regulated genes is not confined to a particular cell type

**Fig 5:**
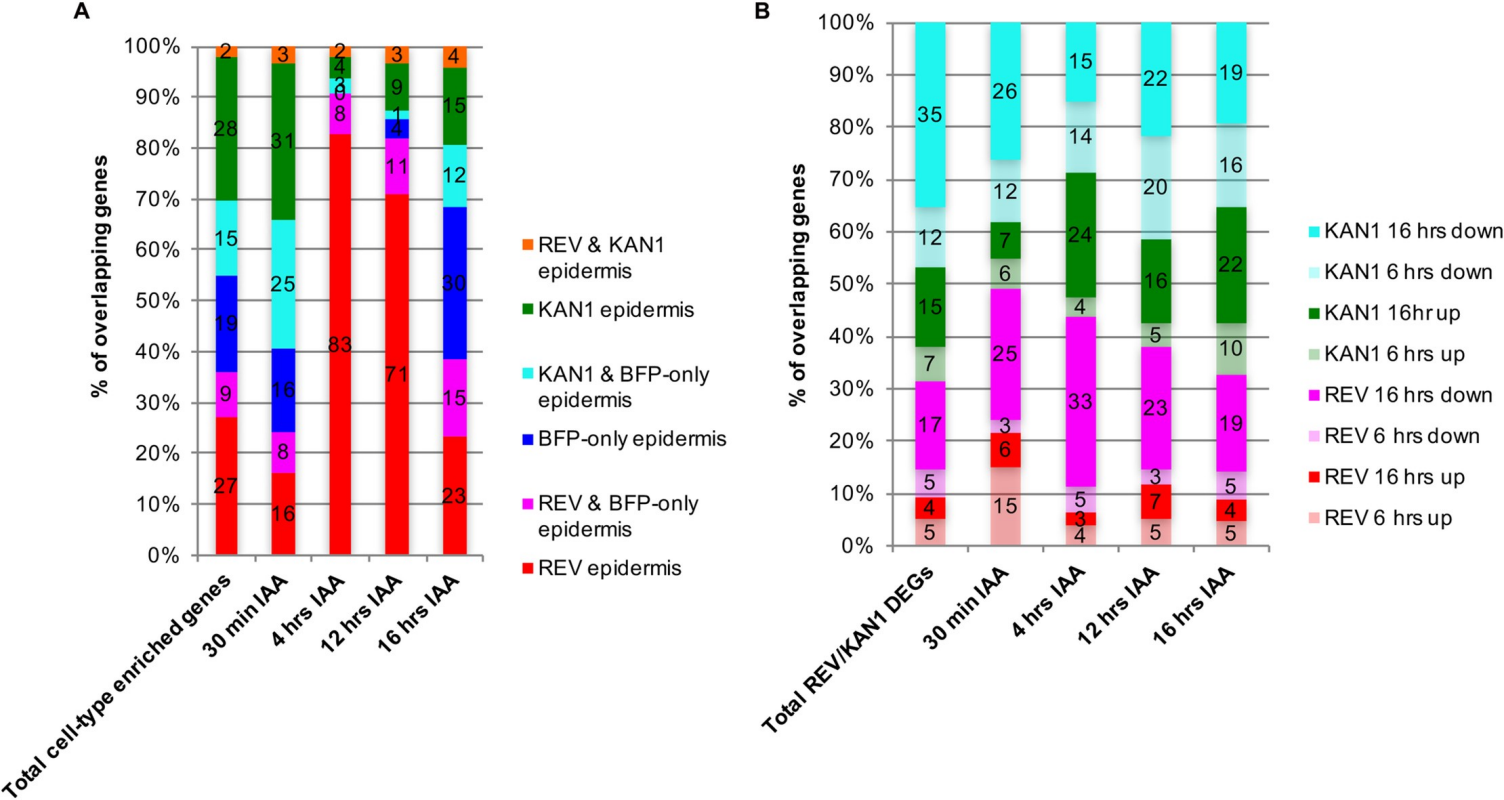
Proportion of auxin responsive genes that are cell-type enriched and responsive to changes in REV and KAN1 expression. (A) Overlap between the set of genes expressed in a cell-type-specific manner and genes responsive to auxin. First bar represents genes enriched in each particular cell-type as a proportion of the total genes that show cell-type specific enrichment (including genes unresponsive to auxin), shown by the color and percentage. Subsequent bars represent the same information but for genes that are up-regulated by auxin at specific time points (see x-axis). Note that the data for auxin up-regulated genes should be compared to the data for all cell-type specific genes (first column). (B). First bar represents genes responsive to REV or KAN1 at different time points (see color legend adjacent to bars) as a proportion of the total number of genes responsive to REV and KAN1 (including genes unresponsive to auxin), shown by the color and percentage. Subsequent bars represent the same information but for genes that are up-regulated by auxin at specific time points (see x-axis). Note that the data for auxin up-regulated genes should be compared to the data for all REV and KAN1 regulated genes (first column).

We next asked whether the expression patterns of identified auxin-regulated genes are specific to particular cell types based on our FACS data. We found that around 60% of genes regulated by auxin at 30 min in *pin1* mutant apices are differentially expressed according to cell type (Table 4). Their overall patterns of expression resembled those found for all genes identified to be differentially expressed according to cell type (Fig 5A). This is somewhat surprising since the DR5 auxin response reporter is restricted spatially in its response to auxin [4, 7, 8]. One likely explanation is that these genes are regulated by other factors in addition to auxin. In contrast, more than 70% of genes up-regulated by auxin at later time points (4 hr and 12 hr) are most highly expressed in the REV domain (Fig 5A). This set includes many cell cycle related genes (*Histones*, *Cyclins*, *Kinesins*, as well as genes involved in other cell cycle related processes), genes involved in floral organ initiation including *ROXY1*, *STYLISH 1*, *SHI-RELATED SEQUENCE 7*, *ANT-LIKE 6*, *TMO6* and *DORNROSCHEN-LIKE (DRNL*), genes involved in organ polarity (*PHABULOSA*, *ZPR3*, *ZPR4*) and two *IAA* genes (*IAA29*, *IAA30*) which lack domain II required for their degradation in presence of auxin (S23 & S24 Tables). Lastly, auxin responsive genes which are up-regulated at 16 hr are over-represented in the set of genes most highly expressed in the BFP-only and BFP-only and REV domain (Fig 5A). This set includes genes involved in establishing the meristem-organ boundary (*CUC2*) as well as genes promoting abaxial identity (*YABBY1*/*FIL*) (S23 & S24 Tables).

**Table 4:** Regulation and expression pattern of auxin responsive genes.

Auxin time points	Total no. of auxin responsive genes	Cell-type specific auxin responsive genes	Auxin responsive genes regulated by REV and/or KAN1
**30 min**	352	208 (59%)	213 (61%)
**4 hrs**	214	146 (68%)	110 (51%)
**12 hrs**	536	280 (52%)	279 (52%)
**16 hrs**	460	246 (53%)	293 (64%)

### Genes regulated by auxin are regulated by REV and KAN1 in diverse ways

Previous studies have suggested a role for KAN1 in the repression of auxin biosynthesis, transport and signalling [4, 9–11, 13]. This conclusion was based on either phenotypic analysis or transcriptomics data reporting the repression of auxin regulated genes by KAN1. For instance, [9,10,13] found that KAN1 repressed the expression of *PIN1* and other related genes encoding auxin transport proteins as well as genes involved in the regulation of these transporters, such as *PID* and *NPY*-related genes. Other auxin-related genes already identified to be repressed by KAN1 include members of the *Aux/IAA* and *ARF* gene families as well as genes involved in auxin synthesis including *YUC* and *TAA* family members. In this study, which focuses on the shoot, we also found many of the same auxin-associated genes to be repressed by KAN1 (S16 Table). In addition, we also provide evidence that many of these genes are expressed at comparatively low levels in the KAN1 SAM domain, as would be expected if KAN1 usually represses these genes during normal development. As for regulation by KAN1, around two thirds of all DEGs regulated by KAN1 6 hrs after induction are repressed and this proportion is similar for the genes in this set that are also induced by auxin at 30 mins (Fig 5B). However for later auxin-regulated genes in this set, e.g. 4 and 12 hrs, this proportion increases, e.g. 78% for 4 hr auxin-induced genes. Genes in this category include genes involved in organ formation such as, *YABBY1*/*FIL* (*AT2G45190*), *ANT-LIKE 6* (AT5G10510), *STYLISH1* (*AT3G51060*), *STYLISH2* (*AT4G36260*), *SHI* (*AT5G66350*), *ROXY1* (*AT3G02000*), *BOP1* (*AT3G57130*), *BOP2* (*AT2G41370*), *DRNL* (*AT1G24590*), *MONOPTEROS* (*AT1G19850*), *ZPR3* (*AT3G52770*), *PHB* (*AT2G34710*), and genes involved in auxin transport such as *PIN1* (*AT1G73590*), *AUX1* (*AT2G38120*), *LIKE AUXIN RESISTANT 2* (*AT2G21050*) (S21 Table), consistent with previous data demonstrating that KAN1 represses auxin transport and organogenesis [4]. For genes regulated by KAN1 at 16 hrs after induction again, most are down-regulated (70%). However this response is reversed for genes in this set that are responsive to auxin at 4 hrs (62% up-regulated). Key genes in this category include *GH3* related genes (*AT4G37390*, *AT2G23170*), which are known to conjugate auxin to amino acids [30], *IAA29* (*AT4G32280*), a known repressors of auxin‐regulated transcription [66, 67] and *HISTIDINE PHOSPHOTRANSFER PROTEIN 6* (*AT1G80100*) (S21 Table), which is a negative regulator of cytokinin signaling. This response therefore suggests that apart from repressing positive regulators of auxin-induced organ growth, KAN1 also indirectly activates genes that nevertheless, also repress pathways that promote organogenesis.

With regard to REV, previous transcriptomics results suggest a role in promoting auxin biosynthesis, transport and signalling [10, 14]. In contrast, detailed analysis of loss of function and ectopic expression induced phenotypes suggest that *REV* and other *HD-ZIPIII* genes act to repress auxin transport and signalling [4, 68]. With respect to genes induced by auxin within 30 mins, our results indicate that a higher proportion of these genes are upregulated by REV at 6 hr compared to all REV DEGs (Fig 5B). Interestingly of the 47 30 min auxin response genes upregulated by REV at 6 hrs, 34 are also downregulated by REV at 16 hrs and of these 34, half (17) belong to cluster 3 from Fig 2A providing further evidence that REV regulates genes associated with auxin response in opposite ways, depending on the time after REV induction.

As discussed above, many of these genes promote glucosinolate production. Other genes transiently upregulated by REV prior to their repression include genes involved in glutathione regulation (Fig 2A). Since both glucosinolates and glutathione are regulated in response to abiotic stress [69], it is possible that transient induction of these genes by REV represents a secondary consequence triggered by stress. However our data also indicates that these genes are normally expressed at relatively low levels in the REV SAM domain compared to the KAN1 domain, consistent with their longer-term repression by REV (S16 Table). This suggests a developmental role for these genes although the exact nature of this role remains unclear. In contrast to this subset of genes, the majority of auxin-regulated genes also regulated by REV at 16 hrs are repressed (Fig 5B), especially genes regulated by auxin after 4 hrs (92%), and include genes involved in organ formation such as *YABBY1*/*FIL*, *ANT-LIKE 6*, *STYLISH1* and *ROXY1* (S11 Fig, S24 Table). Thus these results are consistent with previous results showing that in the SAM, REV represses auxin-dependent organogenesis [4]. Although ultimately repressed by REV, many rapid auxin-induced genes as well as genes slower to respond to auxin, are normally co-expressed with REV according to our data. Although somewhat counterintuitive, this is consistent with the finding that REV itself is indirectly induced by auxin and that its expression domain expands into PIN1 polarity convergences sites soon after their appearance [4].

Overall our data indicate that both REV and KAN1 regulate downstream genes, including auxin-induced genes, in a cell type and gene-specific manner with REV acting to both activate and repress some auxin-regulated genes in a temporally dynamic but indirect fashion. This mode of action appears to contrast with that of WUS, which more directly represses general auxin response in the meristem central zone [70]. Unlike loss of KAN or HD-ZIPIII function however, loss of WUS function does not result in ectopic organogenesis suggesting that the HD-ZIPIII and KAN genes repress organogenesis by acting on specific auxin-regulated targets or pathways and/or by regulating other organogenesis-related genes not associated with auxin. While our study leaves open the question of which genes are involved in such pathways, this work identifies many promising candidates for future investigation.

## Materials and methods

### Plant materials and growth conditions

Plants were grown in soil under continuous light at 22 degrees C. Transgenic *Arabidopsis thaliana* plants were generated by Agrobacterium-mediated transformation. To generate triple marker line for cell-sorting experiments, *pBGW-pREV*::*REV-2YPET;pKAN1*::*KAN1-2GFP* construct was introduced into plants containing *pMOA33-pAtML1*::*mTag-BFP-ER* in *ap1cal* background. To identify downstream targets of REV and KAN1, *pSult-pAtML1*::*GR-LhG4;6OP*::*REVr-2VENUS*, and *pSult-pAtML1*::*GR-LhG4;6OP*::*KAN1-2GFP* were separately transformed into *ap1cal* plants. *pBart-pAtML1*::*GR-LhG4* was introduced into in *ap1cal* plants which were containing *pMOA33-pAtML1*::*mTag-BFP-ER*, to use as a control for RNA-Seq studies involving ectopic expression of REV or KAN1. For knocking-down REV for RNA-Seq experiments, *pAtUBQ10*::*GR-LhG4;6OP*::*miRNA165a* construct was introduced in *ap1cal* background. *pSult-pAtUBQ10*::*GR-LhG4* in *ap1cal* background was used as a control for experiments in which RNA-Seq was done after induction of *pAtUBQ10*::*GR-LhG4;6OP*::*miRNA165a*. *pin1* mutants (also known as *pin1-613*, *pin1-7*, SALK_047613) [8, 71] used for RNA-Seq studies were obtained from the European Arabidopsis Stock Centre (NASC). *p35s*::*GR-REVd* and *p35s*::*KAN1-GR* seeds were a gift from Stephan Wenkel (Copenhagen Plant Science Centre). *rev-1* and *rev phb phv* mutant plants are described in [72] and [73] respectively.

### Constructions of recombinant DNAs

For construction of *pBGW-pREV*::*REV-2YPET;pKAN1*::*KAN1-2GFP* construct, p*REV*::*REV-2Ypet* and *pKAN1*::*KAN1-2*GFP [4] transgenes were combined in T-DNA vector *pBGW* [74] by Gateway technology (Invitrogen). For *pMOA33-pAtML1*::*mTag-BFP-ER* construct, ER-localized *mTag-BFP* [20] was synthesized (Genscript) and cloned as a Bam*HI* fragment downstream of 3.4 kb of the L1-specific *ATML1* gene (*At4g21750*) promoter in *pBJ36* vector, and then whole expression cassette was transferred into T-DNA vector *pMOA33* [75] through Not*I* restriction digestion and ligation. Construction of *pSult-pAtML1*::*GR-LhG4;6OP*::*REVr-2VENUS* and *pSult-pAtML1*::*GR-LhG4;6OP*::*KAN1-2GFP* is explained in [4] and *pAtUBQ10*::*GR-LhG4;6OP*:*mir165a* is explained in [28]. Constructions of *pAtML1*::*GR-LhG4* and *pAtUBQ10*::*GR-LhG4* drivers is described in [4], and these drivers were eventually transferred into the T-DNA vector *pSULT* through Not*I* restriction digestion and ligation [4].

### Dexamethasone treatment of inflorescence meristems

For inducible gene perturbations in the IM, 10 μM DEX solution containing 0.015% Silwet L-77 was applied once to the IM and tissues were collected after required time point for RNA-Seq experiments. For analysing phenotype, the DEX was applied every second day for total 7 days. Inflorescences were then dissected and imaged.

### Protoplasting

Buffer A (10mM KCl, 2mM MgCl_2_, 2mM CaCl_2_, 0.1% BSA, 2mM MES hydrate, 0.6M Mannitol) was filter sterilized and stored at 4°C for long term use. 1.5% Cellulase RS 10 (ONOZUKA, Yakult Pharmaceutical, Japan) and 0.1%Pectolyase Y23 (Kyowa chemical products co. Ltd., Japan) were dissolved into required amount of buffer A by mixing in rotating wheel in dark at 4 degree. About 50 young SAMs of 4–5 week old *ap1cal* plants containing transgenes were quickly harvested and placed in enzyme solution in 70um cell-strainer in a small petridish. After that, the SAMs were chopped into small pieces through sharp blade and incubated in dark at 22°C for 60 minutes on a bench top orbital shaker at 100 rpm. At every 20 minutes, the cell strainer was lifted up and the SAMs were gently pressed against the cell strainer using an autoclaved pestle. The gentle pressing enabled the tissue to loosen and disperse. After that, protoplasts suspension was carefully transferred from the tissue culture dishes to the 15 ml round bottom falcon tube. From this point onwards the protoplasts were kept on ice. The protoplasts were collected by centrifuging at 500g for 7 minutes at 4°C. The supernatant was carefully removed without disturbing the protoplast pellet. To wash the pellet, it was resuspended gently in 5 ml of Buffer A by pipetting. Resuspended protoplasts, were centrifuged at 500 g for 7 mins at 4°C. The supernatant was discarded and the pellet was resuspended in 1 ml of Buffer A. The resuspended pellet suspension was passed through the 40 um cell strainer in a small petri dish and was used for sorting through Fluorescence Activated Cell Sorter (FACS).

### FACS

Samples were sorted by the EMBL flow cytometry core facility using a special setup MoFlo XDP cell sorter from Beckman Coulter equipped with a Coherent Sabr Argon gas laser emitting at 514.5nm (400mW), a Coherent Innova gas laser emitting at 488nm (300mW), and a Coherent Obis 405nm solid state laser (240mW). Forward-(FSC)/side scatter(SSC) and eGFP signals were taken from the 488nm laser (FSC/SSC both 488/10nm band-pass, 505 DCSP beam-splitter; eGFP 510/20nm band-pass + 514nm notch filter). YFP and propidium iodite (PI) signals were recorded from the 514.5nm laser (YFP 545/35nm band-pass + 514nm notch; 605 DCSP beam-splitter; PI 630/40nm band-pass). BFP signals were taken from the 405nm laser (440/40nm band-pass). Spectral overlap was minimal and hence no compensation was necessary. Doubled gating was based on SSC height vs area ratios. For all sorts, FACSFlow (BD) was used as sheath fluid at 30psi and protoplasts were sorted with a 100um nozzle at a frequency of 40kHz. For RNAseq library preparation, 100,000 viable protoplasts were sorted directly into 500 ul RLT buffer (Qiagen) while sample and receptacle cups were kept at 4°C during the whole sort and then immediately shock frozen in liquid nitrogen.

### RNA-extraction and validation

The frozen protoplasts were thawed at 37 degree and then RLT buffer volume was adjusted to 3:1 to volume of protoplasts. After mixing it by vortexing, the mixture was incubated at room temperature for 5 minutes, and then again vortexed vigorously to break down the protoplasts. After that Rneasy Mini kit (Qiagen) was used according to manufacturer’s instruction. An aliquot of RNA was used to check quality and quantity through 2100 Bioanalyzer (Agilent). Another aliquot was used for cDNA preparation using Super script III reverse transcriptase for Q-PCR analysis. Q-PCRs were performed in a StepOne Plus Real Time PCR system thermo cycler (The applied bio systems) using 20μl of PCR reaction contained 10 μL of SYBR Green mix (Roche), 1μl of primer mix (10um), 2μl of diluted cDNA and 7 μl of water. Sequence of primers used is given in S25 Table. Transcript levels were normalized to ACTIN2 transcript levels. Data was analysed using the 2−ΔΔCT method.

### Library preparation

RNA-Seq Libraries for all cell-type experiments and *pin1* mutants were prepared with minimum of 10ng of RNA using ‘NEBNext Ultra RNA Library Prep Kit for Illumina’. For *pAtUBQ10>>miRNA165a* SAM tissue, the libraries were prepared with 1μg of total RNA using ‘TruSeq Stranded mRNA Library Prep Kit from Illumina’.

### Data quality control and sequence alignment

The quality of the sequencing data was assessed using FastQC [76]. PCR primers, bad quality bases (with a Phred score <20) and bases with fluctuating GC content were trimmed off with cutadapt [77]. Genome mapping was performed using TopHat [78], using an index generated from TAIR 10 genome release.

### Differential expression

A gene expression count table was generated from the aligned dataset using HTSeq [79] using TAIR 10 gene annotations and using only the exonic regions of the gene. Differential expression analysis was done using DESeq2 [80] R package and all the genes with an adjusted p-value of < = 0.1 and an absolute log2 fold change of > = 1 were considered as differentially expressed. These set of genes were used in further downstream processing.

### GO enrichment

GO enrichment analysis was performed using topGO [81] Bioconductor package and using “molecular function” Gene Ontology annotations for *A*. *thaliana*. The list of over-represented GO terms were filtered down using the criteria: p-value cut-off: 0.05, minimum number of over represented genes: 5, minimum number of annotated genes: 5

### Clustering

The list of differentially expressed genes from various experiments were clustered based on log2 fold change values using a clustering technique called consensus clustering [82]. In the first step, quantile normalized log2 fold changes were clustered using k-means clustering. In this step, the number of clusters (k), and the total number of genes (n) were sampled randomly. For k, the sampling range was 3 ≤ k ≤√n, while n was resampled with the criteria that the resampled n should not be less than 80% of the total number of genes. For each k-means run, maximum number of iterations and the number of random restarts were set to 500 and 20 respectively. This first step was iterated for 4000 times, and the clustering consensus (similarity) between two genes in the input list was calculated as:

*similarity* = *Nr*. *of times two genes appear in the same cluster* / *Nr*. *of times two genes were sampled together*

This consensus clustering matrix was filtered down to exclude genes with a very weak similarity. This filtering was done using personalized page rank algorithm [83] to model the clustering consensus between a given gene and its neighbours. In this step, each gene was selected as the starting node and personalized page rank was computed for rest of the genes in the consensus matrix. These page rank values were sorted and top 65% of a genes’ neighbours were retained in the consensus matrix. In the final step of the clustering process, the filtered consensus matrix was clustered using Infomap clustering algorithm [84] implemented in igraph R package [85] with the number of random trials parameter was set to 1000.

### Western blot

Total proteins were extracted from 5 days old seedling tissues of *rev-1*, Col-0 and *rev phb phv* triple mutant in protein extraction buffer (400m M sucrose, 50m M Tris-Cl pH 7.5, 10% glycerol, 2.5 mM EDTA). The protein extract was transferred to fresh 1.5 ml tube and centrifuged at 13000 rpm for 5 min to pellet down the debris. The supernatant was transferred to a fresh 1.5 ml tube, and an aliquot of 2 ul was taken out in a separate tube for the estimation of protein concentration by Bradford assay. To the 50 ug of total protein, appropriate amount of 6x of sample loading buffer (200 mM Tris-Cl, pH 6.8, 400 mM dithiothreitol, 4% SDS, 0.025% Bromphenol Blue, 20% glycerol) was added and boiled for 5 min before loading on SDS-PAGE. Proteins were then transferred to Hybond ECL nitrocellulose membrane (GE Healthcare) at 100 mA for 1 hr in transfer buffer (Tris (7.56 g), glycine (47 g), 20% methanol in 2.5 liters) in Mini blot protein gel apparatus (GE Healthcare). The membrane was then incubated for overnight in 10 ml of blocking buffer (5% non-fat dry milk in Tris-buffered saline and 0.05% Tween 20) at 4 degree C with shaking. The blocking reagent was removed, and the affinity-purified primary antibody (Anti-REV) diluted (1:1000) in 10 ml of blocking buffer with 0.05% Tween 20 was added and incubated for 2 h with shaking at room temperature. The membrane was then washed with 10 ml of wash buffer (Tris-buffered saline and 0.05% Tween 20) for thrice, 5 min each. The secondary antibody, conjugated with horseradish peroxidase diluted (1:10,000) in 15 ml of blocking buffer with 0.05% Tween 20, was added and incubated for 1 h with shaking at room temperature. The membrane was washed with 15 ml of wash buffer for five times at room temperature. The working solution of substrate was prepared by mixing peroxide solution and luminol/enhancer solution in 1:1 ratio, and the blot was incubated in that working solution for 5 min. The blot was then removed from the working solution and covered with plastic wrap in a cassette and exposed to x-ray film for different times.

### Dexamethasone and Cycloheximide treatment for Q-PCR

The Q-PCR results shown in Fig 3 are obtained from 4 days seedling tissues of *p35s*::*GR-REVd* and *p35s*::*KAN1-GR* lines in wild-type (Ler) background. For Q-PCR results shown in S10 Fig, SAMs from transgenic line containing *p35s*::*GR-REVd* in *ap1cal* background were used. Whole seedlings (for seedling experiment) or whole shoots (for *ap1cal* SAMs) were immersed in solutions containing 10 μM dexamethasone in 0.1% ethanol or 0.1% ethanol (mock) or 10 μM dexamethasone + 20 μM cycloheximide or 0.1% ethanol (mock)+ 20 μM cycloheximide solutions. Seedling tissues were treated for 3 hrs and SAMs were treated for 2 hrs. After that vegetative meristem and first pair of leaves were dissected and collected from seedlings tissues, whereas SAMs were collected from *ap1cal* line containing *p35s*::*GR-REVd*. Three biological replicates were collected for each treatment, each line and each tissue type. RNA extraction from seedling tissue was done using RNeasy Mini kit (Qiagen) following manufacturer’s instruction, whereas ISOLATE II RNA Mini kit (Bioline) was used for RNA extraction from SAM tissue. SensiFAST cDNA Synthesis Kit (for SAM tissue) and Super script III reverse transcriptase (for seedling tissue) were used for cDNA synthesis. Q-PCRs were performed in a StepOne Plus Real Time PCR system thermo cycler, Applied Bio Systems (ABI) (for seedling tissue) and LightCycler 480 II, ABI (for SAMs) according to the manufacture’s instructions. Sequence of primers used is given in S25 Table. Transcript levels were normalized to *ACTIN2* transcript levels. Data was analysed using the 2−ΔΔCT method.

#### Validation of spatial expression patterns of selected DEGs in SAM

*pREV*::*REV-2YPET;pPIN1*::*PIN1-CFP* transgenic line has been previously described [4]. For preparing DNA constructs for validation of spatial expression patterns of *AT5G20740*, *AT1G71520* and *AT3G04290* genes, full-length promoters of these genes were amplified using infusion primers (*AT5G20740* FP- GCGGCCGCATGCATATGCTTACTTTTACTACGATTACGAGGAGATTG, *AT5G20740* RP- TGTTGGATCCAAGCTTTATCTCTCGGGTTAGAGTAAGAGACC; *AT1G71520* FP- GCGGCCGCATGCATATGACTATATAGTCTTCATTTCTATTCATTTCTATTCATTTTATG, *AT1G71520* RP- TGTTGGATCCAAGCTTGTTTTTGGGTGGATGAAGATGAGATTA; *AT3G04290* FP- GCGGCCGCATGCATATGGGAGTAGTTGGTCTGCATGTGTATG, *AT3G04290* RP-TGTTGGATCCAAGCTTTGTTATGGACAGAGAATGGGACTAGAT). These promoters were then individually cloned in front of *NLS-TdTomato* in *pBJ36* vector through infusion cloning after digesting the vector with BamH*I*. Later whole expression cassette containing *promoter*::*NLS-TdTomato-pea3A-terminator* was transferred into T-DNA vector *pMOA34* [75] through Not*I* restriction digestion and ligation. These constructs were then individually transformed into transgenic line containing *pBGW-pREV*::*REV-2YPET;pKAN1*::*KAN1-2GFP* construct. For *AT1G14600* reporter, full-length promoter and genomic region was amplified using *AT1G14600* FP-CGATAAGCTTGGATCAGGTATTAGAGTTAGTAAAATCAAAG and *AT1G14600* RP AGCCGCAGCAGGATCGCTATGGAGTAGAGAAAAGG and then cloned in frame with 2YPET in *pBJ36* vector through infusion cloning. Later, the whole expression cassette was transferred into the T-DNA vector *pMOA34* [75] through Not*I* restriction digestion and ligation and then transformed into transgenic line containing *pPIN1*::*PIN1-GFP* [86]. *DRN* translational reporter (*pDRN*::*DRN-GFP*) is reported in [87]. This construct was transformed into *pREV*::*REV-2YPET;pPIN1*::*PIN1-CFP* [4] transgenic line. For *DRNL* reporter, its expression cassette, which consists of its 5,650 bp upstream and 3,822 bp downstream regulatory sequences [88] was hooked to the *GR-LHG4* in *pENTER L5L2* vector (Invitrogen). Another entry vector *pENTER L1R5* containing *6xOP*::*mCherry-ER*, *pENTER L5L2* containing *DRNL* expression cassette with *GR-LHG4*, and Gateway destination vector *pBGW* (Invitrogen) were used in Gateway LR reaction (Invitrogen) to produce final construct, *pBGW-DRNL expression cassette*::*GR-LHG4;6OP*::*mCherry-ER*. This constructs was transformed into transgenic line containing *pPIN1*::*PIN1-GFP* [86]. *ANT* reporter, *pANT*::*ANT-VENUS*, has previously been described [89]. *pHAT3*::*VENUS-HAT3* [28] and *pKAN1*::*KAN1-2xGFP* [4] were combined in T-DNA vector p*KGW* [74] by Gateway technology (Invitrogen) for generation of a double marker transgenic line *pHAT3*::*VENUS-HAT3;pKAN1*::*KAN1-2xGFP*. A 4687 bp genomic fragment of the *DOF5*.*8* (*AT5G66940*) gene was amplified using forward primer GGACCCATGAAAGCTTCTTTCTTTGCT and reverse primer CGCTACGTAGTCTCCAGACACGA and then fused as a translational fusion to *YPET* to create *pDOF5*.*8*::*DOF5*.*8-YPET*. A double marker line was generated by transforming the *pDOF5*.*8*::*DOF5*.*8-YPET* into a *pPIN1*::*PIN1-GFP* transgenic line. For *AS2* reporter, *pAS2*::*2VENUS-AS2* translational fusion was constructed by putting 5037 bp *AS2* promoter sequence, using FP acgCGTTTCGTCTAATTCACTTCTCTGTGA and RP ggtaccTTTAATGACTTGAAAATGGAGTTT-TTC, in front of *2VENUS* fused to the *AS2* coding sequence using which was amplified by FP ggatccATGGCATCTTCTTCAACAAACTCACCATG and RP ggatccccAGACGGATCAACAG-TACG-GCGACCAT. The construct was transformed into the *pPIN1*::*PIN1-CFP* transgenic line.

## Supporting information

S1 Fig Flow cytometric analysis of protoplasts derived from *ap1cal* triple marker line, containing *pREV*::*REV-2YPET*, *pKAN1*::*KAN1-2GFP*, *pAtML1*::*mTag-BFP-ER* reporters, stained with Propidium Iodide (PI). (A) Total cell population resolved by Forward and Side Scatter, (B) All single protoplasts from the protoplast gate in (A) as determined by Area vs Height pulse shape analysis on Side Scatter signals, (C) Distribution of all singlet protoplasts from the region marked in panel (B) for presence of PI and BFP signals. (D) Distribution of BFP- PI- protoplasts for YFP and GFP signals from plot (C). (E) Distribution of BFP+ PI- protoplasts for presence of YFP and GFP signals from plot (C). All fluorescent values (BFP, GFP and YFP) are plotted as log-height intensity values.
(PDF)

S2 Fig Analysis of gene expression for sorted protoplasts from triple marker line containing *pREV*::*REV-2YPET*, *pKAN1*::*KAN1-2GFP*, *pAtML1*::*mTag-BFP-ER* reporters. (A-C) Q-PCR analysis for selected marker genes expressed in sorted protoplasts used for preparing RNA-Seq libraries. The values are normalized to internal reference *ACT2*. (D) Principle Component Analysis of RNA-Seq data obtained from triple marker line containing *pREV*::*REV-2YPET*, *pKAN1*::*KAN1-2GFP*, *pAtML1*::*mTag-BFP-ER* reporters.
(PDF)

S3 Fig Comparison of cell-type specific genes with relative cell-type specific genes described in [18]. Overlap between the set of genes expressed in a cell-type-specific manner in this study and in a previous study by [18]. First bar represents genes enriched in a particular cell-type as a proportion of the total genes that show cell-type specific enrichment, shown by the color and percentage. Subsequent bars represent the same information but for genes that are up-regulated in particular cell-type (see x-axis). Note that the data for FIL, CLV3, KAN1, LAS domains should be compared to the data for all cell-type specific genes (first column).
(PDF)

S4 Fig Validation of cell-type specific expression pattern of selected genes. (A) Selected genes and their enriched expression patterns according to FACS-based RNA-seq data. (B-O) Confocal longitudinal optical sections of WT meristems expressing fluorescent markers corresponding to genes in (A) as well as reference genes. (B) Expression of *pREV*::*REV-2YPET* together with *pPIN1*::*PIN1-CFP* for comparison to other markers shown in other panels of this figure. (C) Expression of *pANT*::*ANT-2VENUS together with pPIN1*::*PIN1-GFP*. (D) and (G) *NLS-TdTomato*-based transcriptional reporter (see Methods) for *Arabidopsis* gene *AT5G20740* in comparison to *pREV*::*REV-2YPET* and *pKAN1*::*KAN1-2GFP* respectively (same optical section but different channels). (E & H) are same optical sections but different channels, in which *NLS-TdTomato*-based reporter (see Methods) for *AT1G71520* is shown in comparison to *REV-2YPET* (E), *and pKAN1*::*KAN1-GFP is shown in comparison to pREV*::*REV-2YET* (H). Note, *AT1G71520* and *KAN1* have very simialr expression pattern in (E) and (H), respectively. (F & I) *NLS-TdTomato*-based transcriptional reporter (see Methods) for *AT3G04290* together with *pREV*::*REV-2YPET* and *pKAN1*::*KAN1-2GFP* respectively. (J) *YPET*-based translational reporter for *AT1G14600* (see methods) together with *pPIN1*::*PIN1-GFP*. (K) *YPET*-based translational reporter for *DOF5*.*8* (see methods) together with *pPIN1*::*PIN1-GFP*. (L) *pHAT3*::*VENUS-HAT3* together with *pKAN1*::*KAN1-2GFP*. (M) *pDRN*::*DRN-GFP* together with *pREV*::*REV-2YPET* and *pPIN1*::*PIN1-CFP*. (N) *mCherry-ER* based transcriptional reporter for *DRNL* together with *pPIN1*::*PIN1-GFP*. (O) *pAS2*::*2VENUS-AS2* together with *pPIN1*::*PIN1-CFP*. Note expression of *pAS2*::*2VENUS-AS2* in SAM centre as well as periphery. Arrowheads in different panels indicate incipient primordia marked by high *pPIN1*::*PIN1-G(C)FP* signal. Scale bars: 20 μm (B); 100 μm (C); 40 μm (D), (E), (G) and (H); 30 μm (F) and (I); 15 μm (J), (K), (M), (L), (N) and (O).
(PDF)

S5 Fig Phenotype of transgenic lines used for RNA-Seq. (A) SAM of *ap1cal* plants, which were used as a genetic background for transgenic line generation. (B) *ap1cal* SAM after 2 weeks induction of *pAtUBQ10>>miRNA165a*. Note that the meristems have been converted to radialised organs (C) a*p1cal* SAM after 1 week induction of *pAtML1>>REVr-2VENUS*. Note meristems have become “pin” shaped due to the arrest of organogenesis. (D) *ap1cal* SAM after 1 week induction of *pAtML1>>KAN1-2GFP*. As for (C), organogenesis has been arrested.
(PDF)

S6 Fig Flow cytometric analysis of *A*. *thaliana* protoplasts from various transgenic lines used for RNA-Seq. (A) Distribution of all singlet protoplasts from the *pAtML1*::*mTag-BFP-ER* line for presence of PI and BFP signals. (B-C) Distribution of all singlet protoplasts from *pAtML1>>KAN1-2GFP* line for presence of PI and GFP signals. (D-E) Distribution of all singlet protoplasts from *pAtML1>>REVr-2VENUS* line for presence of PI and YFP signals. (B & D) after 6hr induction of transgene, (C & E) after 16 hrs induction of transgene. All fluorescent values (BFP, GFP and YFP) are plotted as log-height intensity values.
(PDF)

S7 Fig Response of known target genes to REV and KAN1 induction in transgenic lines used for RNA-Seq. (A, B and C) Q-PCR analysis after induction of transgenes at various time points. The values are normalized to internal reference *ACT2*. (D) Western blot analysis to check specificity of anti-REV antibodies in crude total protein extracts from seedling tissues of indicated genotypes. (E) Western blot analysis for REV protein using anti-REV antibodies on *ap1cal* SAM tissue after induction of *pAtUBQ10>>miRNA165a* at specified time points.
(PDF)

S8 Fig Co-regulation of common targets of REV and KAN1. (A) Co-regulation of all the common targets of REV and KAN1 after 6 hr induction of *pAtML1>>REVr-2VENUS* and *pAtML1>>KAN1-2GFP*. (B) Co-regulation of all the common targets of REV and KAN1 after 16 hr induction of *pAtML1>>REVr-2VENUS* and *pAtML1>>KAN1-2GFP*.
(PDF)

S9 Fig Comparison of gene families regulated in both shoot (this study) and leaf. Green text denotes positive regulation, red text denotes negative regulation. * = 0.1>p>0.05, ** = Only in Q-PCR, *** = Down in shoot, up in leaf, **** = Up in shoot, down in leaf, ^#^ = opposite regulation at early and late time point, ^1^ = identified in two leaf studies, ^2^ = identified in three leaf studies, ^3^ = identified in four leaf studies
(PDF)

S10 Fig Q-PCR analysis of selected genes in presence and absence of cycloheximide. Q-PCR analysis of selected genes in SAMs of *p35s*::*GR-REVd* line after 2 hrs of mock (ethanol) or dex treatment (A), or mock (ethanol)+cycloheximide or dex+cycloheximide treatment (B). ZPR1 and ZPR3 are known REV direct targets, so they are used as positive controls. *ACT2* was used as an internal reference gene. N = 3, * = p<0.05, ** = p<0.01, *** = p<0.001.
(PDF)

S11 Fig Cell type expression and regulatory data for selected genes responsive to IAA. Numbers in the table indicate the number of genes common between row and column. Name of the developmental related genes which belongs to respective intersection is given in the bracket (along with the no.). Color of the gene name indicates cell-type specific enrichment. REV epidermis (red), KAN1 epidermis (green), REV epidermis & BFP-only epidermis (purple), KAN1 epidermis & BFP-only epidermis (light blue), BFP-only epidermis (dark blue), REV epidermis & KAN1 epidermis (orange) and un-enriched (back).
(PDF)

S1 Table Epidermal cell-type enriched genes. A gene is considered as enriched in a epidermal cell-type if it has log2 FC>1 with p<0.05 in that cell-type against any of the other two epidermal cell-types.
(XLSX)

S2 Table Sets of genes shown in the venn diagram from Fig 1H. (XLSX)

S3 Table Sub-epidermal cell-type enriched genes. A gene is considered as enriched in a sub-epidermal cell-type if it has log2 FC>1 with p<0.05 in that cell-type against any of the other two sub-epidermal cell-types.
(XLSX)

S4 Table CPEGs (cell population differentially expressed genes) identified by [18], which are relevant to this study and used for comparison in this study. CPEGs in [18], are genes that show elevated expression in one cell type (target) compared with all other cell types (reference) in the comparison group B, which includes LAS, CLV3, FIL, and KAN1 cell-types, (1.5 fold change and p<0.05).
(XLSX)

S5 Table Comparison of previously published expression data with FACS-based profiling results. During organ development REV expression extends from the central meristem region into incipient primordia [86], hence we divide the REV expression domain into two regions corresponding to the meristem center and primordia.
(XLSX)

S6 Table Details of clustering analysis of cell-type profilling experiment, and GO enrichment analysis of the identified clusters. (XLSX)

S7 Table Details of DEGs identified after induction of *pAtML1>>REVr-2VENUS*. (XLSX)

S8 Table Details of DEGs identified after induction of *pAtML1>>KAN1-2GFP*. (XLSX)

S9 Table Details of DEGs identified after induction of *pAtUBQ10>>miRNA165a*. (XLSX)

S10 Table Comparative analysis of DEGs identified at 6 hrs or 16 hrs induction of *pAtML1>>REVr-2VENUS* and *pAtML1>>KAN1-2GFP*. (XLSX)

S11 Table Details of clustering analysis performed on REV/KAN1 targets shown in Fig 2A and GO enrichment analysis of the identified clusters. (XLSX)

S12 Table Comparison of DEGs identified after *pAtML1>>REVr-2VENUS pAtML1>>KAN1-2GFP* induction with previously published studies in which REV or KAN1 were induced in seedlings. (XLSX)

S13 Table Number of genes in each epidermal cell-type and their overlap with REV/KAN1 regulated genes. (XLSX)

S14 Table Details of cluster 3 genes from Fig 2A, which are expressed more highly outside REV domain, and their GO enrichment analysis. (XLSX)

S15 Table List of genes upregulated by REV at 16 hrs and down regulated by KAN1 and have enriched expression in both REV and BFP-only cells. (XLSX)

S16 Table Log2 FC after REV/KAN1 induction, cell-type expression and regulation by auxin for selected development related genes. (XLSX)

S17 Table Comparative analysis of DEGs identified at 6 hrs induction of *pAtML1>>REVr-2VENUS* and *pAtML1>>KAN1-2GFP*. (XLSX)

S18 Table Comparative analysis of DEGs identified at 16 hrs induction of *pAtML1>>REVr-2VENUS* and *pAtML1>>KAN1-2GFP*. (XLSX)

S19 Table Log2 FC after REV/KAN1 induction, cell-type expression and regulation by auxin for selected development related genes which are co-regulated by REV and KAN1. (XLSX)

S20 Table List of genes up-regulated by auxin (Log2 FC >2 at 30 min) that are functionally characterized in the literature (according to TAIR). (XLSX)

S21 Table Overlap analysis between different sets of DEGs identified in different RNA-Seq experiments. (XLSX)

S22 Table Differentially expressed genes after auxin application on *pin1* meristems, their clustering analysis and GO enrichment analysis of identified clusters. (XLSX)

S23 Table Log2 FC after REV/KAN1 induction, cell-type expression and regulation by auxin for selected development related genes which were identified as DEGs in RNA-Seq experiment in *pin1*. (XLSX)

S24 Table Development related genes up-regulated by auxin at particular time point, their expression changes (Lg2 FC) after REV/KAN1 induction and cell type expression. (XLSX)

S25 Table Sequence of the primers used in qPCR experiments in this study. (XLSX)

S26 Table Underlying numerical data for all the graphs presented in this paper. (XLSX)

S1 Text Description of clusters identified in cell-type profiling (Fig 1I). (PDF)

S1 References Bibliography for the S5 Table. (PDF)
