# Supplementary figures and images for: An integrated analysis of cell-type specific gene expression reveals genes regulated by REVOLUTA and KANADI1 in the Arabidopsis shoot apical meristem
Source: PLoS Genet. 2020 Apr 15;16(4):e1008661. doi: 10.1371/journal.pgen.1008661 (PMC7266345; doi:10.1371/journal.pgen.1008661)

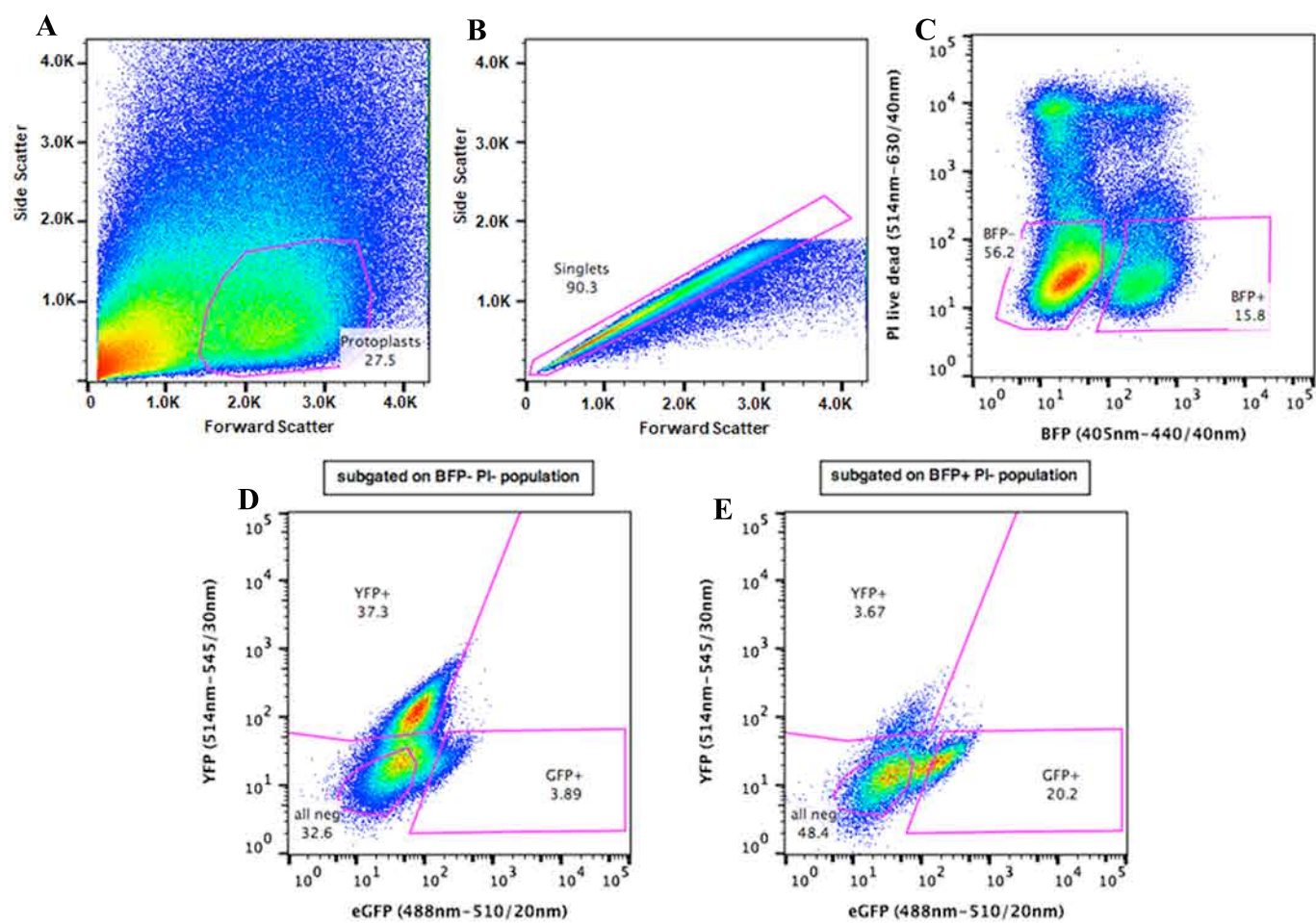

S1 Fig.

Supplement: S1 Fig — (A) Total cell population resolved by Forward and Side Scatter, (B) All single protoplasts from the protoplast gate in (A) as determined by Area vs Height pulse shape analysis on Side Scatter signals, (C) Distribution of all singlet protoplasts from the region marked in panel (B) for presence of PI and BFP signals. (D) Distribution of BFP- PI- protoplasts for YFP and GFP signals from plot (C). (E) Distribution of BFP+ PI- protoplasts for presence of YFP and GFP signals from plot (C). All fluorescent values (BFP, GFP and YFP) are plotted as log-height intensity values. (PDF) [file pgen.1008661.s001.pdf]

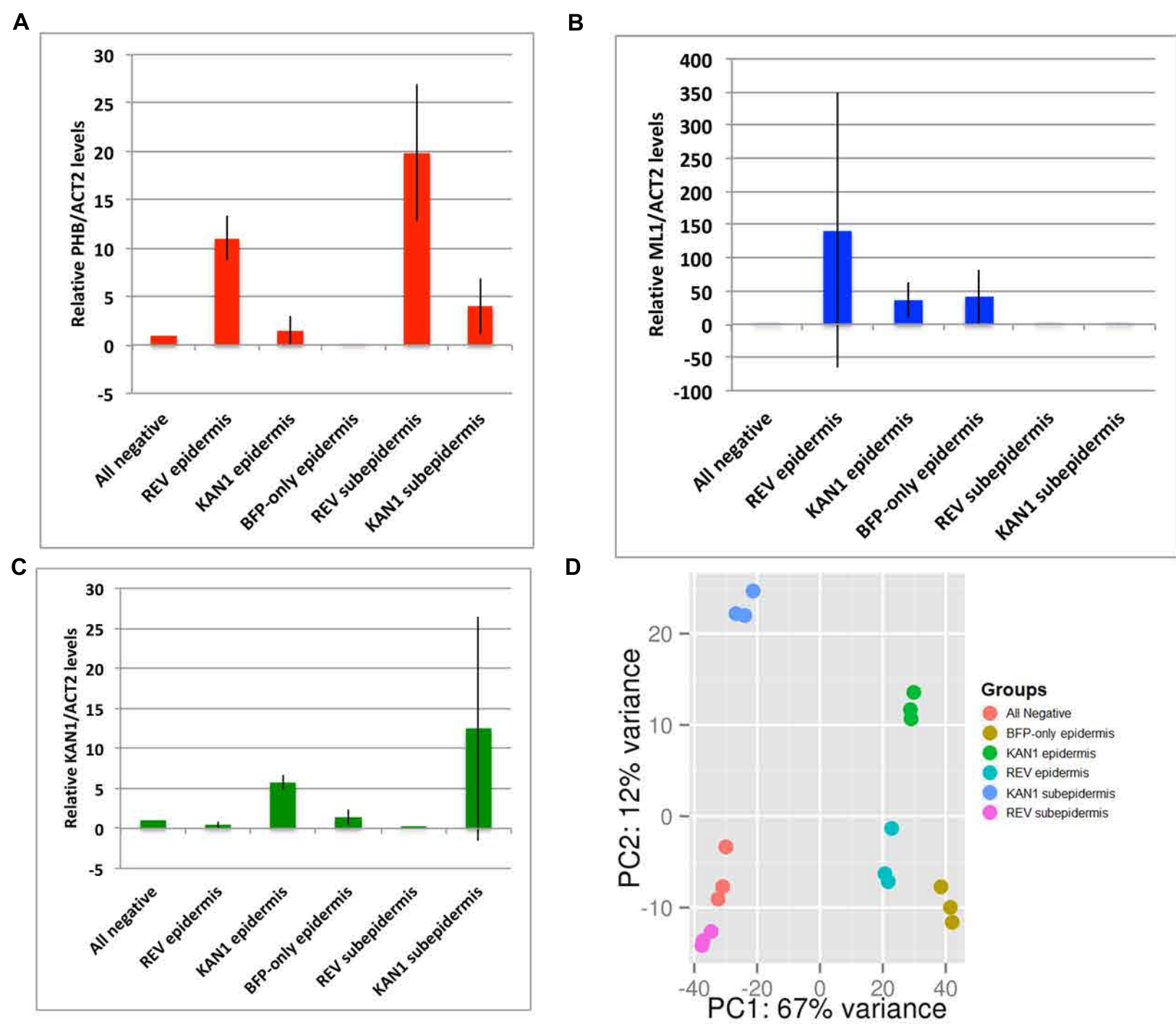

S2 Fig.

Supplement: S2 Fig — (A-C) Q-PCR analysis for selected marker genes expressed in sorted protoplasts used for preparing RNA-Seq libraries. The values are normalized to internal reference ACT2. (D) Principle Component Analysis of RNA-Seq data obtained from triple marker line containing pREV::REV-2YPET, pKAN1::KAN1-2GFP, pAtML1::mTag-BFP-ER reporters. (PDF) [file pgen.1008661.s002.pdf]

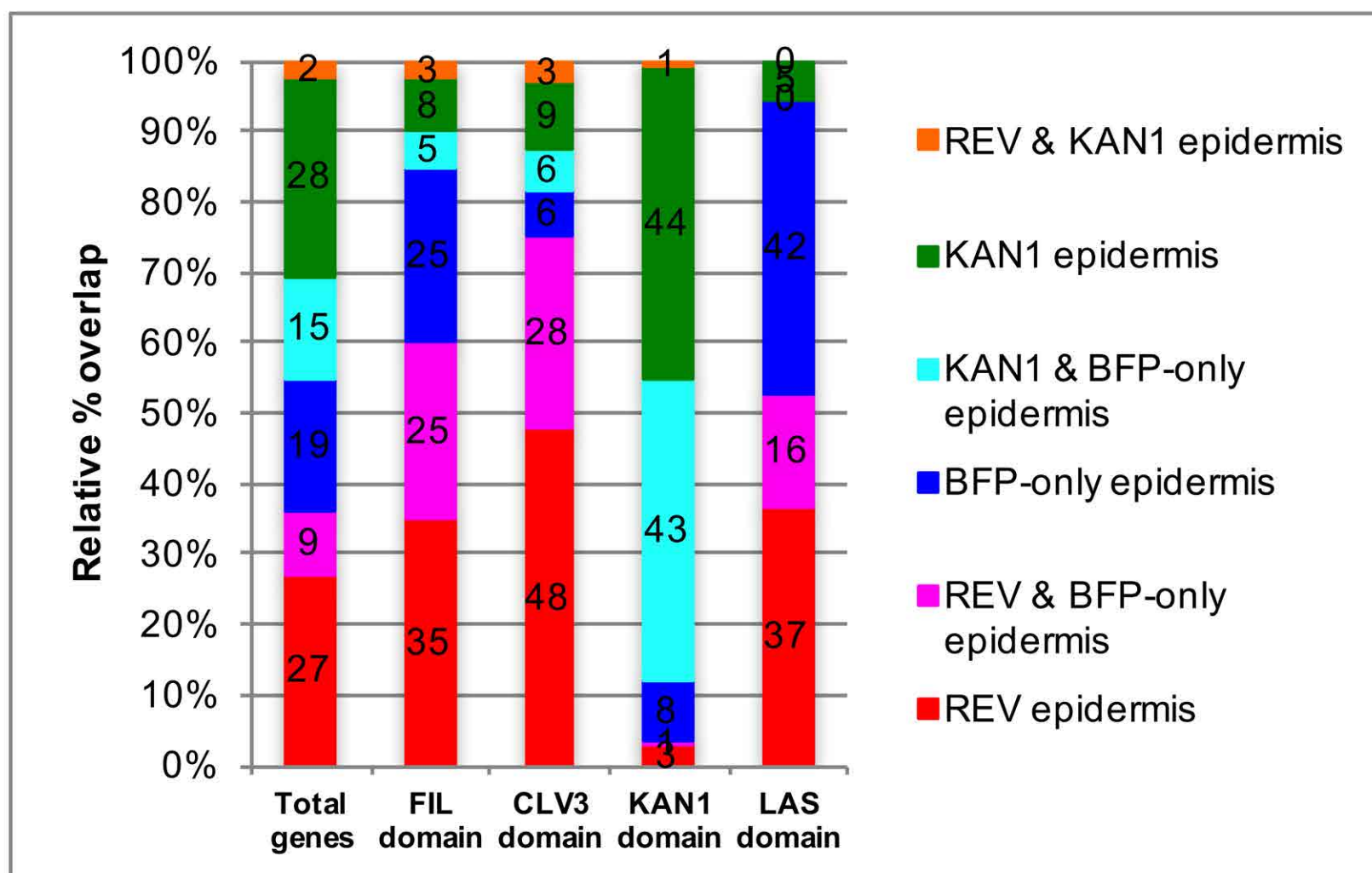

S3 Fig.

Supplement: S3 Fig — Overlap between the set of genes expressed in a cell-type-specific manner in this study and in a previous study by [18]. First bar represents genes enriched in a particular cell-type as a proportion of the total genes that show cell-type specific enrichment, shown by the color and percentage. Subsequent bars represent the same information but for genes that are up-regulated in particular cell-type (see x-axis). Note that the data for FIL, CLV3, KAN1, LAS domains should be compared to the data for all cell-type specific genes (first column). (PDF) [file pgen.1008661.s003.pdf]

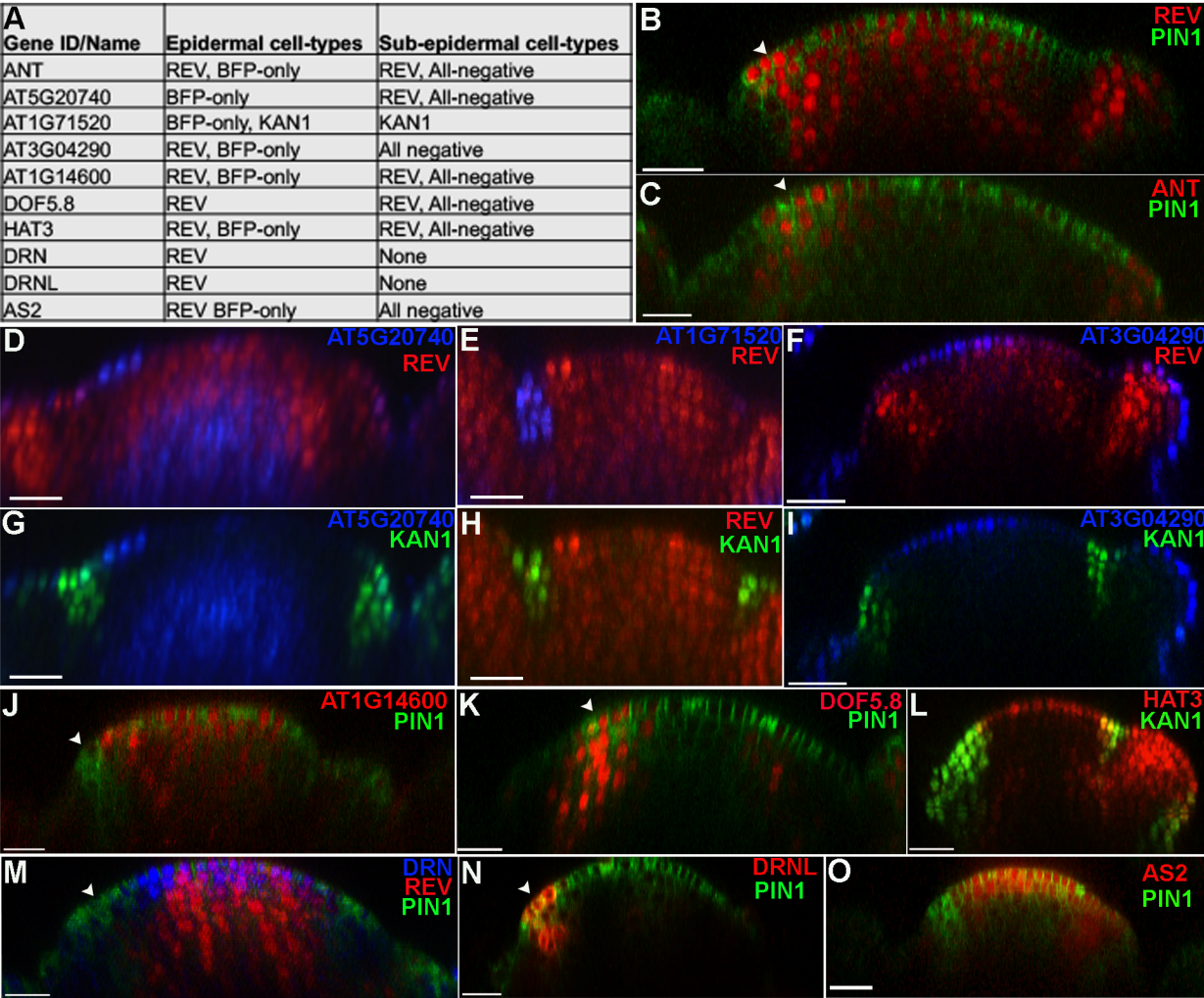

Fig S4

Supplement: S4 Fig — (A) Selected genes and their enriched expression patterns according to FACS-based RNA-seq data. (B-O) Confocal longitudinal optical sections of WT meristems expressing fluorescent markers corresponding to genes in (A) as well as reference genes. (B) Expression of pREV::REV-2YPET together with pPIN1::PIN1-CFP for comparison to other markers shown in other panels of this figure. (C) Expression of pANT::ANT-2VENUS together with pPIN1::PIN1-GFP. (D) and (G) NLS-TdTomato-based transcriptional reporter (see Methods) for Arabidopsis gene AT5G20740 in comparison to pREV::REV-2YPET and pKAN1::KAN1-2GFP respectively (same optical section but different channels). (E & H) are same optical sections but different channels, in which NLS-TdTomato-based reporter (see Methods) for AT1G71520 is shown in comparison to REV-2YPET (E), and pKAN1::KAN1-GFP is shown in comparison to pREV::REV-2YET (H). Note, AT1G71520 and KAN1 have very simialr expression pattern in (E) and (H), respectively. (F & I) NLS-TdTomato-based transcriptional reporter (see Methods) for AT3G04290 together with pREV::REV-2YPET and pKAN1::KAN1-2GFP respectively. (J) YPET-based translational reporter for AT1G14600 (see methods) together with pPIN1::PIN1-GFP. (K) YPET-based translational reporter for DOF5.8 (see methods) together with pPIN1::PIN1-GFP. (L) pHAT3::VENUS-HAT3 together with pKAN1::KAN1-2GFP. (M) pDRN::DRN-GFP together with pREV::REV-2YPET and pPIN1::PIN1-CFP. (N) mCherry-ER based transcriptional reporter for DRNL together with pPIN1::PIN1-GFP. (O) pAS2::2VENUS-AS2 together with pPIN1::PIN1-CFP. Note expression of pAS2::2VENUS-AS2 in SAM centre as well as periphery. Arrowheads in different panels indicate incipient primordia marked by high pPIN1::PIN1-G(C)FP signal. Scale bars: 20 μm (B); 100 μm (C); 40 μm (D), (E), (G) and (H); 30 μm (F) and (I); 15 μm (J), (K), (M), (L), (N) and (O). (PDF) [file pgen.1008661.s004.pdf]

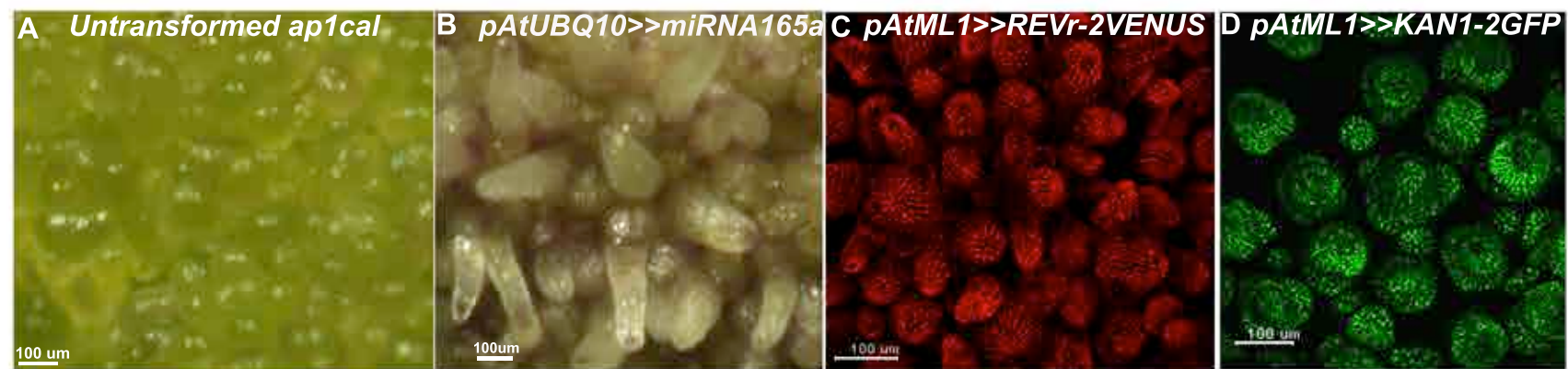

S5 Fig.

Supplement: S5 Fig — (A) SAM of ap1cal plants, which were used as a genetic background for transgenic line generation. (B) ap1cal SAM after 2 weeks induction of pAtUBQ10>>miRNA165a. Note that the meristems have been converted to radialised organs (C) ap1cal SAM after 1 week induction of pAtML1>>REVr-2VENUS. Note meristems have become “pin” shaped due to the arrest of organogenesis. (D) ap1cal SAM after 1 week induction of pAtML1>>KAN1-2GFP. As for (C), organogenesis has been arrested. (PDF) [file pgen.1008661.s005.pdf]

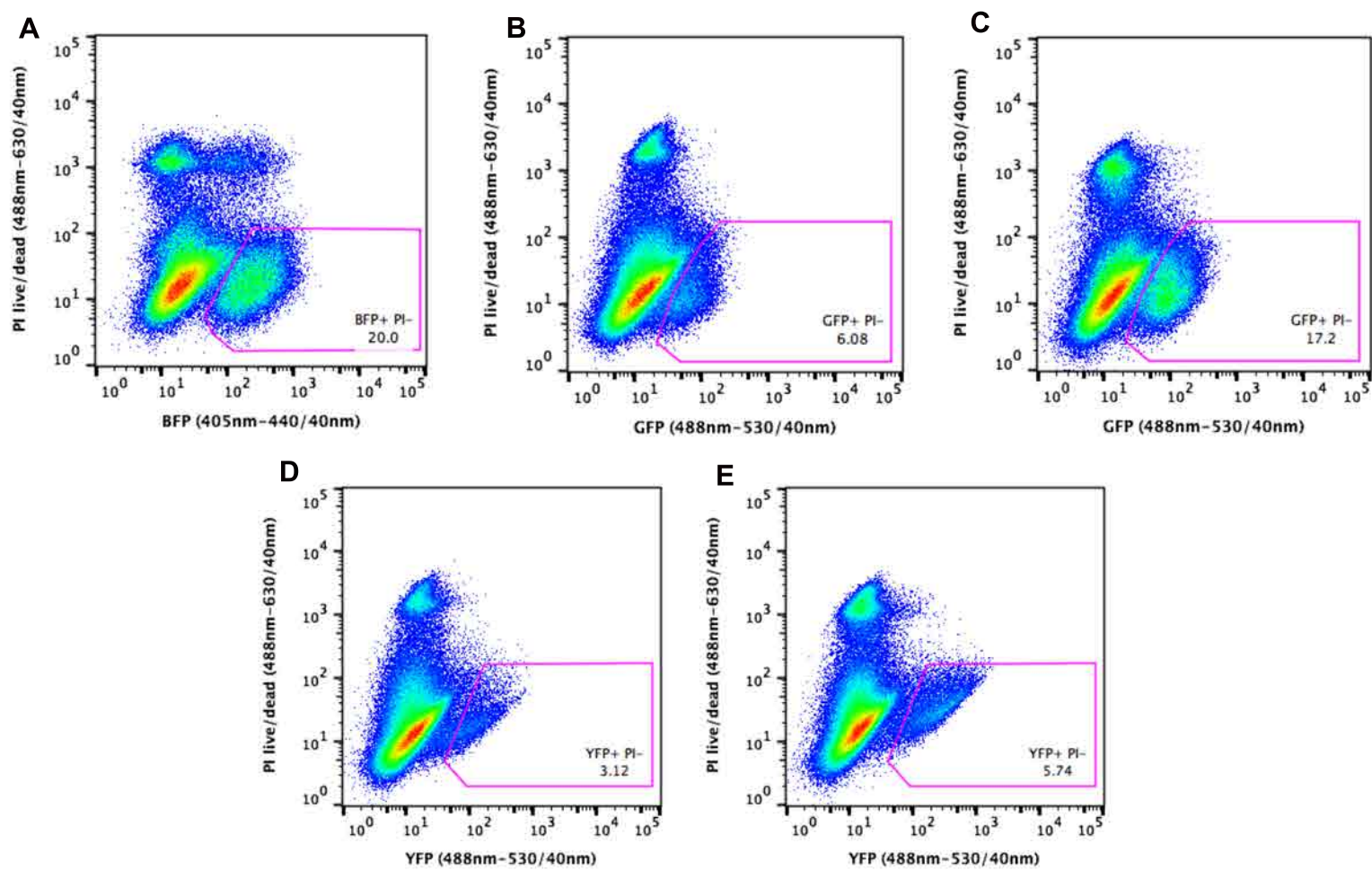

S6 Fig.

Supplement: S6 Fig — (A) Distribution of all singlet protoplasts from the pAtML1::mTag-BFP-ER line for presence of PI and BFP signals. (B-C) Distribution of all singlet protoplasts from pAtML1>>KAN1-2GFP line for presence of PI and GFP signals. (D-E) Distribution of all singlet protoplasts from pAtML1>>REVr-2VENUS line for presence of PI and YFP signals. (B & D) after 6hr induction of transgene, (C & E) after 16 hrs induction of transgene. All fluorescent values (BFP, GFP and YFP) are plotted as log-height intensity values. (PDF) [file pgen.1008661.s006.pdf]

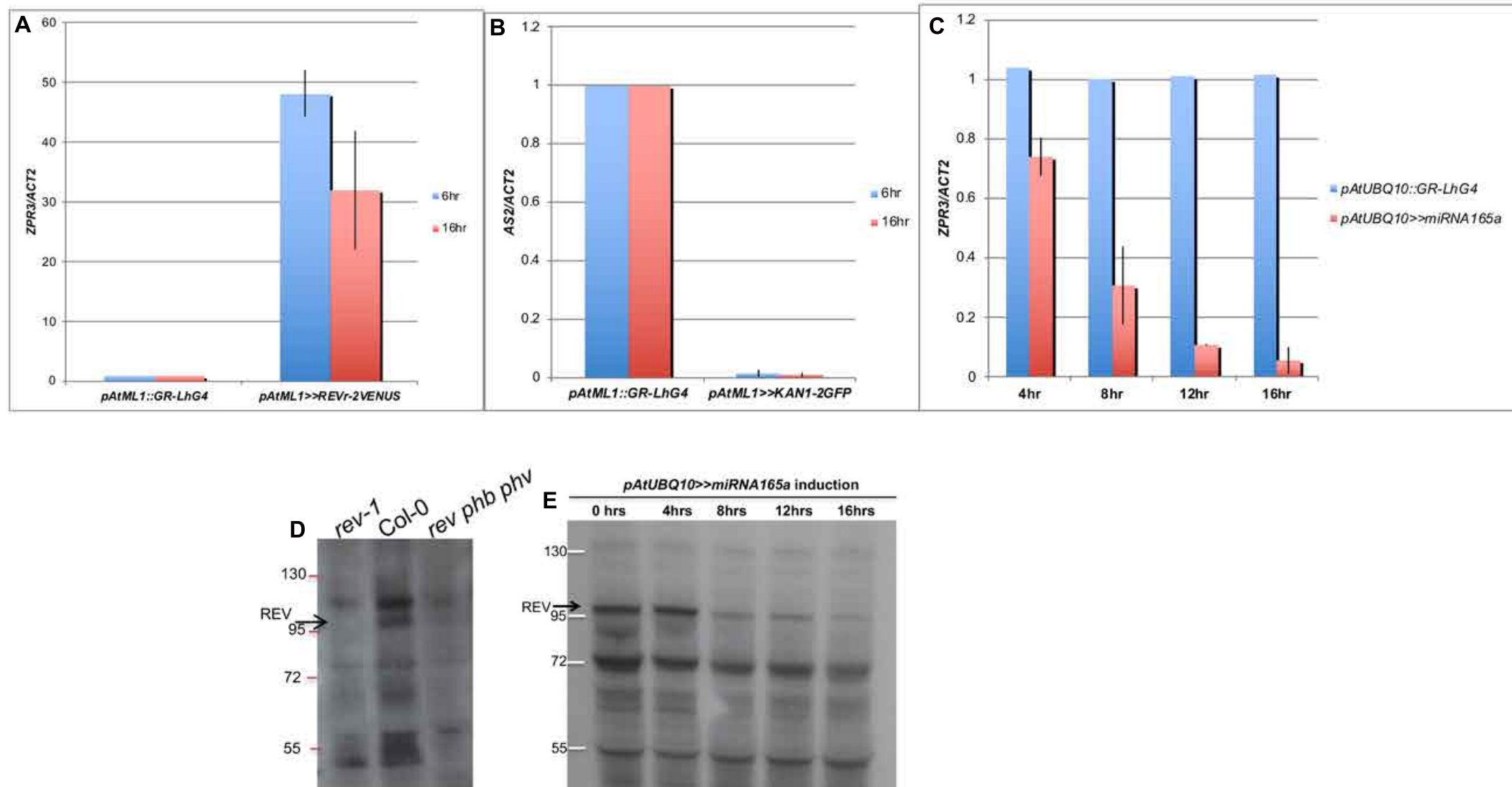

S7 Fig.

Supplement: S7 Fig — (A, B and C) Q-PCR analysis after induction of transgenes at various time points. The values are normalized to internal reference ACT2. (D) Western blot analysis to check specificity of anti-REV antibodies in crude total protein extracts from seedling tissues of indicated genotypes. (E) Western blot analysis for REV protein using anti-REV antibodies on ap1cal SAM tissue after induction of pAtUBQ10>>miRNA165a at specified time points. (PDF) [file pgen.1008661.s007.pdf]

A

REV & KAN1 common targets at 6hr

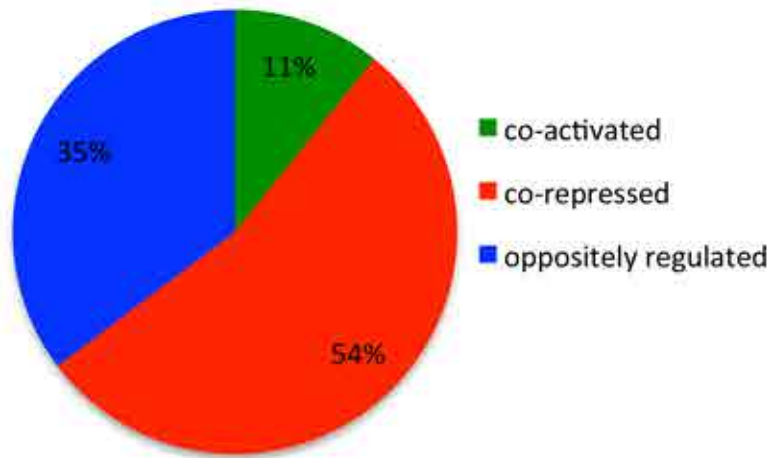

B

REV & KAN1 common targets at 16 hr

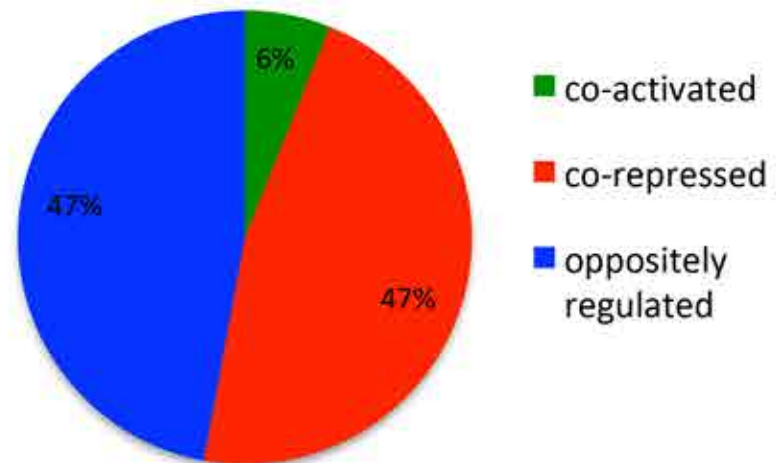

S8 Fig.

Supplement: S8 Fig — (A) Co-regulation of all the common targets of REV and KAN1 after 6 hr induction of pAtML1>>REVr-2VENUS and pAtML1>>KAN1-2GFP. (B) Co-regulation of all the common targets of REV and KAN1 after 16 hr induction of pAtML1>>REVr-2VENUS and pAtML1>>KAN1-2GFP. (PDF) [file pgen.1008661.s008.pdf]

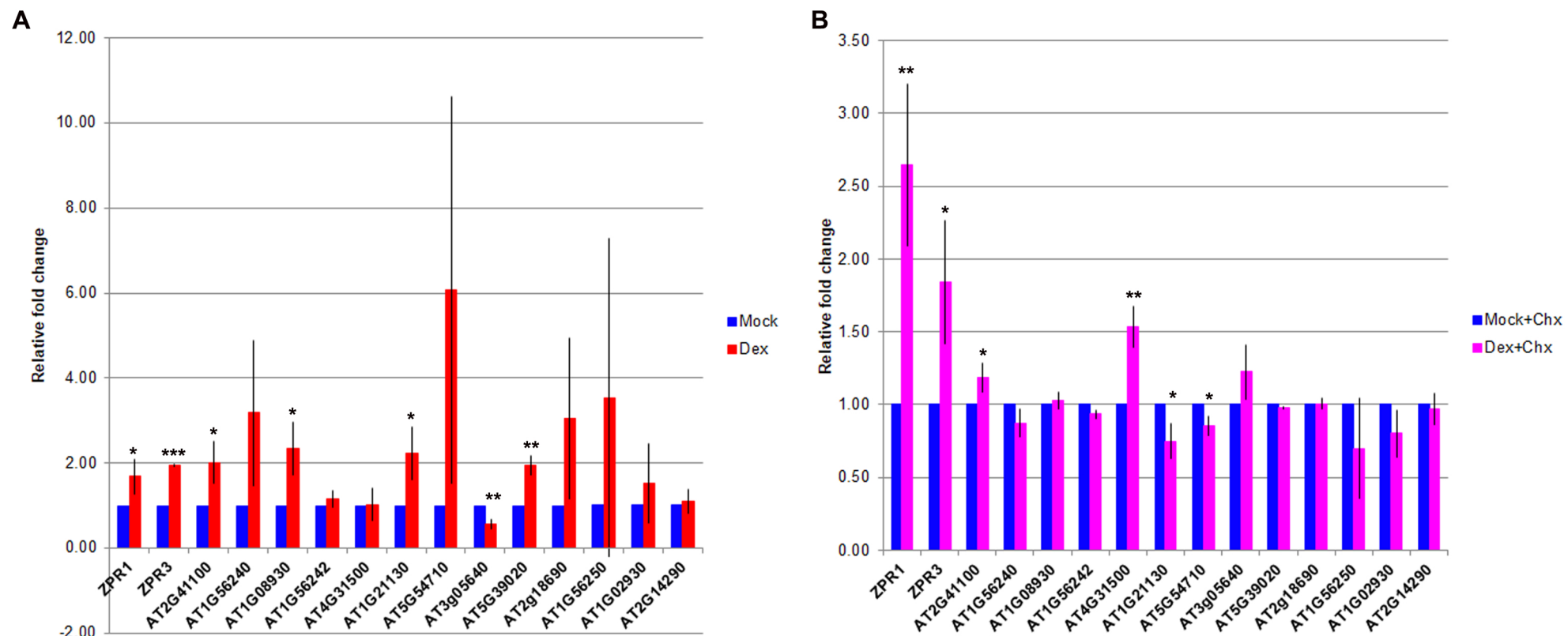

Fig S10.

Supplement: S10 Fig — Q-PCR analysis of selected genes in SAMs of p35s::GR-REVd line after 2 hrs of mock (ethanol) or dex treatment (A), or mock (ethanol)+cycloheximide or dex+cycloheximide treatment (B). ZPR1 and ZPR3 are known REV direct targets, so they are used as positive controls. ACT2 was used as an internal reference gene. N = 3, * = p<0.05, ** = p<0.01, *** = p<0.001. (PDF) [file pgen.1008661.s010.pdf]
