# Supplementary material for: An integrated analysis of cell-type specific gene expression reveals genes regulated by REVOLUTA and KANADI1 in the Arabidopsis shoot apical meristem
Source: PLoS Genet. 2020 Apr 15;16(4):e1008661. doi: 10.1371/journal.pgen.1008661 (PMC7266345; doi:10.1371/journal.pgen.1008661)
Supplement: S9 Fig — Green text denotes positive regulation, red text denotes negative regulation. * = 0.1>p>0.05, ** = Only in Q-PCR, *** = Down in shoot, up in leaf, **** = Up in shoot, down in leaf, # = opposite regulation at early and late time point, 1 = identified in two leaf studies, 2 = identified in three leaf studies, 3 = identified in four leaf studies (PDF) [file pgen.1008661.s009.pdf]

S9 Fig.

Gene family	Regulation by REV in both shoot and leaf	Regulation by REV only in shoot (this study)	Regulation by REV only in leaf [1]	Regulation by KAN1 in both shoot and leaf	Regulation by KAN1 only in shoot (this study)	Regulation by KAN1 only in leaf [1, 9, 10, 13]
Class II HD-ZIPs	HAT1, HAT3, HAT14, HAT22*, ATHB4**	HAT9, HAT17	HAT2, HAT4**	HAT1[1], HAT22[1]	HAT3, HAT9, HAT14#	HAT2[2], ATHB22
FANTASTIC FOUR (FAF)	FAF1	FAF2, FAF3, FAF4		FAF1***	FAF2	
LITTLE ZIPPER (ZPR)	ZPR1, ZPR3	ZPR4			ZPR3	
CLAVATA3/ESR-RELATED	CLE41	CLE21, CLE27, CLE44		CLV1, CLE43	CLE9, CLE13, CLV3, CLE12, CLE14,CLE16, CLE19, CLE21, CLE27, CLE42, CLE44	CLV2
INDETERMINATE DOMAIN (IDD)		IDD12, IDD15, IDD16, JKD	IDD4, IDD11,	IDD5*,****, JKD[3],****, IDD14[1], IDD15	IDD2	IDD4[1]
BEL LIKE HOMEODOMAIN (BLH)		BLH3, BLH5, BLH6, BLH8		BEL1, BLH3*[2], BLH4, BLH7[1],	BLH2, BLH5, BLH8	
YELLOW STRIPE-LIKE (YSL)		YSL1, YSL3,	YSL5*	YSL1[1], YSL3*[2], YSL5	YSL4, YSL7	
KANADI (KAN)	KAN3*	KAN1, KAN2		KAN2*[1], KAN3[1]	KAN4	
ZINC FINGER PROTEINS (ZFP)	ZFP8	ZFP7		ZFP7[1], ZFP8[2]	ZFP1	ZFP4[1]
ARGONOUTE (AGO)		AGO3, AGO5	AGO7 (ZIPPY), AGO10 (ZLL)	AGO10 (ZLL)	AGO2, AGO5, AGO7 (ZIPPY), AGO9	
Auxin biology families	SAUR79	SAUR41, SAUR54, IAA30, NPY1, SAUR35, SAU36, SAUR42, SAUR50, SAUR70, YUC2, YUC4, PIN3, PIN6, PIN7, ARF19, GH3.1, GH3.2, GH3.3, GH3.5, GH3.6, GH3.10, GH3.12, GH3.17, IAA1, IAA2, IAA4, IAA14, IAA17, IAA19, IAA22, IAA29, LAX3	SAUR44, SAUR65, YUC5, TAA1, ARF3*, SAUR6, SAUR51	SAUR6[1], SAUR41, YUC8, TAA1, PIN1, PIN4[3], PIN7, PIN LIKES1, PIN LIKES3[2], NPY3, AUX1[1], ARF4[1], GH3.10, IAA2[2], IAA7[1], IAA13[1], IAA14[1], IAA16[1], LAX1, NPY1	SAUR5, SAUR50, SAUR69, SAUR74, YUC3, YUC5, TMO6, GH3.2, GH3.3, GH3.4, IAA1, IAA29, SAUR12, SAUR42, SAUR51, SAUR59, SAUR72, YUC1, YUC11, PIN4, PIN5, ARF5 (MP), ARF6, ARF16, GH3.12, IAA6, IAA8, IAA15, IAA31, LAX2, ROXY1	ARF10, SAUR1[2], SAUR14[1], SAUR15, SAUR16[1], SAUR19, SAUR20, SAUR21, SAUR22, SAUR49, SAUR52, SAUR53, SAUR62, SAUR64, SAUR68, SAUR78, PIN3, YUC2, YUC6, GH3.3[1], GH3.5[1], GH3.6[1], IAA3[1], IAA4, IAA17, IAA18, IAA22, NPY5, ROXY2[1]
