# Supplementary material for: An integrated analysis of cell-type specific gene expression reveals genes regulated by REVOLUTA and KANADI1 in the Arabidopsis shoot apical meristem
Source: PLoS Genet. 2020 Apr 15;16(4):e1008661. doi: 10.1371/journal.pgen.1008661 (PMC7266345; doi:10.1371/journal.pgen.1008661)
Supplement: S11 Fig — Numbers in the table indicate the number of genes common between row and column. Name of the developmental related genes which belongs to respective intersection is given in the bracket (along with the no.). Color of the gene name indicates cell-type specific enrichment. REV epidermis (red), KAN1 epidermis (green), REV epidermis & BFP-only epidermis (purple), KAN1 epidermis & BFP-only epidermis (light blue), BFP-only epidermis (dark blue), REV epidermis & KAN1 epidermis (orange) and un-enriched (back). (PDF) [file pgen.1008661.s011.pdf]

S11 Fig. Expression dynamics of important IAA responsive genes

	IAA up 30 min (318)	IAA up 4hr (117)	IAA up 12 hr (291)	IAA up 16 hr (138)	IAA down 30 min (34)	IAA down 4hr (97)	IAA down 12hr (245)	IAA down 16hr (322)
**REV up 6 hr & 16 hr**	5 (HAT22, HAT3, HAT1)	0	6 (ZPR3, ZPR4, LOG7, RAX1)	3 (LOG7)	2	0	3	7 (PYR1)
**REV up 6hr**	42 (IAA30, OBP1)	3 (WUS, IAA30, FTM1)	4 (IAA30)	4 (IAA30)	0	7	5 (CYP79B2, CYP79B3)	22 (CYP79B2, SUR2, CYP79B3)
**REV up 16hr**	15 (BOP1, DRN, UNICORN, ATHB13)	2 (SHI-RELATED 7)	7 (SHI-RELATED 7, BOP1, BOP2)	3 (BOP1)	1 (BOP2)	2	3	11
**REV down 6 hr & 16 hr**	2 (ROXY1)	3 (ROXY1)	3 (AIL6, FIL)	4 (ROXY1, FIL)	2	0	13 (ATHB5)	5 (GH3.6)
**REV 6 hr down**	6 (IAA4, GH3.5)	1 (IAA29)	3 (PHB, IAA4)	4 (IAA29)	2	4	20	13 (KNAT3)
**REV down 16 hr**	75 (ACL5, TMO5, TMO6, DOF5.8, ATHB8, IAA1, IAA2, IAA19, GH3.2, GH3.3, AHP6)	23 (STY1, GH3.2, GH3.3, AHP6, TMO5-LIKE 1)	43 (TMO5, TMO6, STY1, ROW1, DOF5.8, IAA2, GH3.2, GH3.3, AHP6)	24 (ACL5, STY1, DOF5.8, IAA1, IAA19, TMO5-LIKE1, YAB5)	18 (ROPGEF 5)	31 (ACL5, LAX3)	65 (SPL3, CYP79B2, METHYL ESTRASE 3, FAF4, CYP79B3, AMI1)	80 (LONGIFOLIA2, CYP79B2, SUR2, CYP79B3, METHYL ESTRASES 2, AIR3)
**KAN1 up 6 & 16 hr**	8 (IAA1, GH3.2, AHP6)	3 (GH3.2, AHP6)	6 (GH3.2, AHP6)	12 (IAA1)	0	3	15 (METHYL ESTRASES)	11 (METHYL ESTRASE 2)
**KAN1 up 6 hr**	10 (TMO6, ATHB8)	0	3 (TMO6)	3	2	1	5	8 (ROTUNDIFOLIA LIKE 8)
**KAN1 up 16 hr**	13 (GH3.3)	16 (IAA29, GH3.3)	26 (GH3.3)	21 (IAA29, TRANSPARENT TESTA 4, ERECTA-LIKE 2)	7	5	26	15
**KAN1 down 6 & 16 hr**	30 (HAT22, ROXY1, DRN, OBP1, IAA26, HAT3, IAA13, BOP1, PIN1, AUX1, LAX1)	7 (ROXY1, STY1, PIN1, AUX1)	18 (AIL6, RAX1, STY1, LAX2, HAT3, IAA13, BOP1, BOP2, AUX1, LOG7, FIL, STY2)	17 (ROXY1, STY1, LAX2, BOP1, BOP2, AUX1, PIN1, LOG7, STY2, FIL)	2 (BOP2)	7	26 (PME3)	39 (BLH1)
**KAN1 down 6 hr**	7 (DRNL)	4 (DRNL)	21 (DRNL, ZPR3, PHB, SHI-RELATED 5, SHI)	7 (DRNL, SHI, PAN)	2 (HAN)	0	3	5 (SAUR51)
**KAN1 down 16 hr**	52 (TMO6, HAT1, CKX3, IAA2, UNICORN)	5	25 (DOF5.8, TMO6, IAA2)	12 (DOF5.8, CKX5, ARR3)	5 (ATHB21)	26 (CKX3)	55 (METHYL ESTRASES 10)	77 (LONGIFOLIA2, SUR2, KNAT3, METHYL ESTRASES 10)
