# Supplementary material for: An integrated analysis of cell-type specific gene expression reveals genes regulated by REVOLUTA and KANADI1 in the Arabidopsis shoot apical meristem
Source: PLoS Genet. 2020 Apr 15;16(4):e1008661. doi: 10.1371/journal.pgen.1008661 (PMC7266345; doi:10.1371/journal.pgen.1008661)
Supplement: S1 Text — (PDF) [file pgen.1008661.s038.pdf]

Supplementary Text S1

Description of clusters identified in cell-type profiling (Fig. 1I)

Cluster 6: cluster 6 represents genes repressed in both REV and KAN1 epidermis compared to BFP-only epidermis and amongst the genes within this group, the GO term organ development is significantly enriched. Examples include LATERAL ORGAN BOUNDARIES (LOB), LATERAL SUPPRESSOR (LAS), which is involved in the initiation of axillary meristems, NGATHAs, AP2/B3-like transcriptional factor family proteins which are involved in gynoecium development, PRESSED FLOWER, a homeodomain protein which regulates lateral axis-dependent development of flowers, and PIN4, an auxin efflux carrier.

Cluster 5: Similar to cluster 6, this group of genes are repressed in both REV and KAN1 domains, although the fold change of most of the genes in this cluster is milder than cluster 6. The developmentally related GO terms enriched in this cluster include unidimensional cell growth, multidimensional cell growth, cell tip growth, cell size regulation. Apart from cell growth, auxin transport and auxin response are other important GO terms significantly enriched in this cluster.

Cluster 4: In this cluster most of the genes are repressed in REV domain compared to both KAN1 and BFP-only domains. 2 GO terms significantly enriched in this cluster are cell growth and auxin synthesis. Many of the genes from this cluster are homologues of genes from cluster 5 and 6, such as EXPANSIN, LONGIFOLIA, NGATHAs LIKES, TRANSPARENT TESTA, AGAMOUS LIKEs, BEL1 LIKEs. Four members of the zinc finger homeodomain (ZF-HD) transcriptional factor family are also present in this cluster.

Cluster 2: Compared with BFP-only domain, expression of these genes is repressed in REV domain and up-regulated in KAN1 domain. In other words, expression of these genes is present in a gradient being high in KAN1 domain to medium level in BFP-only cells, and low expression levels in REV domain. Interestingly, of the GO terms related with organ development, auxin synthesis and auxin response are enriched in this cluster.

Cluster 1: It represents genes having enriched expression in REV epidermis, compared with both BFP-only and KAN1 epidermis. More than 10% of the genes from this cluster are associated with cell-cycle. Meristem function and organ development are two other GO terms enriched in this cluster. Genes involved in meristem function include class III HD ZIPs (PHB, ATHB15, ATHB8) and their targets (LITTLE ZIPPERs), RAX1 (involved in axillary meristem formation), CLV3 & STIMPY (involved in homeostasis of stem cells), cytokinin signaling pathway genes (LOG1, LOG7, AHK1, ARR1). In the organ development GO category, many genes promoting organ growth such as ANT and ANT likes, PUCHI, DRN, DRN-Like, LEAFY, BOP2, STY1, SHI, ACL5, SEP3, ROXY1, HAT3, JLO and auxin signaling components such as YUC4, PIN1, PIN6, AUX1, LAX2, IAA13, IAA29, IAA30, IAA31, NPY1, DOF transcription factors (TMO6, DOF4.6, DOF5.8, DOF6).
