# Supplementary material for: An integrated analysis of cell-type specific gene expression reveals genes regulated by REVOLUTA and KANADI1 in the Arabidopsis shoot apical meristem
Source: PLoS Genet. 2020 Apr 15;16(4):e1008661. doi: 10.1371/journal.pgen.1008661 (PMC7266345; doi:10.1371/journal.pgen.1008661)
Supplement: S1 References — (PDF) [file pgen.1008661.s039.pdf]

Supplementary references

1. Otsuga D, DeGuzman B, Prigge MJ, Drews GN, Clark SE. REVOLUTA regulates meristem initiation at lateral positions. Plant J. 2001;25(2):223–36. PubMed PMID: 8538741
2. McConnell JR, Emery J, Eshed Y, Bao N, Bowman J, Barton MK. Role of PHABULOSA and PHAVOLUTA in determining radial patterning in shoots. Nature. 2001;411(6838):709–13. PubMed PMID: 15988559
3. Heisler MG, Ohno C, Das P, Sieber P, Reddy GV, Long JA, et al. Patterns of auxin transport and gene expression during primordium development revealed by live imaging of the Arabidopsis inflorescence meristem. Curr Biol. 2005;15(21):1899–911. PubMed PMID: 16271866
4. Long JA, Moan EI, Medford JI, Barton MK. A member of the KNOTTED class of homeodomain proteins encoded by the STM gene of Arabidopsis. Nature. 1996;379(6560):66–9. PubMed PMID: 21700457
5. Nole-Wilson S, Tranby TL, Krizek BA. AINTEGUMENTA-like (AIL) genes are expressed in young tissues and may specify meristematic or division-competent states. Plant Mol Biol. 2005;57(5):613–28. PubMed PMID: 15845148
6. Prasad K, Grigg SP, Barkoulas M, Yadav RK, Sanchez-Perez GF, Pinon V, et al. Arabidopsis PLETHORA transcription factors control phyllotaxis. Curr Biol. 2011;21(13):1123–8. PubMed PMID: 11169198
7. Anderson GH, Veit B, Hanson MR. The Arabidopsis AtRaptor genes are essential for post-embryonic plant growth. BMC Biol. 2005;3:12. PubMed PMID: 11395776
8. Franco-Zorrilla JM. AtREM1, a Member of a New Family of B3 Domain-Containing Genes, Is Preferentially Expressed in Reproductive Meristems. Plant Physiology. 2002;128:418–27. PubMed PMID: 11842146
9. Furutani M, Kajiwara T, Kato T, Treml BS, Stockum C, Torres-Ruiz RA, et al. The gene MACCHI-BOU 4/ENHANCER OF PINOID encodes a NPH3-like protein and reveals similarities between organogenesis and phototropism at the molecular level. Development. 2007;134(21):3849–59. PubMed PMID: 17913786
10. Xing S, Rosso MG, Zachgo S. ROXY1, a member of the plant glutaredoxin family, is required for petal development in Arabidopsis thaliana. Development. 2005;132(7):1555–65. PubMed PMID: 15728668
11. Yadav RK, Girke T, Pasala S, Xie M, Reddy GV. Gene expression map of the Arabidopsis shoot apical meristem stem cell niche. Proc Natl Acad Sci USA.

2009;106(12):4941-6. PubMed PMID: 19258454
12. Fletcher JC, Brand U, Running MP, Simon R, Meyerowitz EM. Signaling of cell fate decisions by CLAVATA3 in Arabidopsis shoot meristems. Science. 1999;283(5409):1911–4. PubMed PMID: 10082464
13. Anderson GH, Alvarez NDG, Gilman C, Jeffares DC, Trainor VCW, Hanson MR, et al. Diversification of Genes Encoding Mei2-Like RNA Binding Proteins in Plants [Internet]. Plant Molecular Biology. 2004;54:653–70. PubMed PMID: 15356386
14. Xu L, Xu Y, Dong A, Sun Y, Pi L, Xu Y, et al. Novel as1 and as2 defects in leaf adaxial-abaxial polarity reveal the requirement for ASYMMETRIC LEAVES1 and 2 and ERECTA functions in specifying leaf adaxial identity. Development. 2003;130(17):4097–107. PubMed PMID: 12874130
15. Borghi L, Bureau M, Simon R. Arabidopsis JAGGED LATERAL ORGANS is expressed in boundaries and coordinates KNOX and PIN activity. Plant Cell. 2007;19(6):1795-808. PubMed PMID: 17557810
16. Carles CC, Choffnes-Inada D, Reville K, Lertpiriyapong K, Fletcher JC. ULTRAPETALA1 encodes a SAND domain putative transcriptional regulator that controls shoot and floral meristem activity in Arabidopsis. Development. 2005;132(5):897–911. PubMed PMID: 15673576
17. Hibara K-I, Karim MR, Takada S, Taoka K-I, Furutani M, Aida M, et al. Arabidopsis CUP-SHAPED COTYLEDON3 regulates postembryonic shoot meristem and organ boundary formation. Plant Cell. 2006;18(11):2946–57. PubMed PMID: 17122068
18. Durfee T, Roe JL, Sessions RA, Inouye C, Serikawa K, Feldmann KA, et al. The F-box-containing protein UFO and AGAMOUS participate in antagonistic pathways governing early petal development in Arabidopsis. Proc Natl Acad Sci USA. 2003;100(14):8571–6. PubMed PMID: 12826617
19. Norberg M, Holmlund M, Nilsson O. The BLADE ON PETIOLE genes act redundantly to control the growth and development of lateral organs. Development. 2005 May;132(9):2203–13. PubMed PMID: 15800002
20. Hepworth SR, Zhang Y, McKim S, Li X, Haughn GW. BLADE-ON-PETIOLE-dependent signaling controls leaf and floral patterning in Arabidopsis. Plant Cell. 2005;17(5):1434–48. PubMed PMID: 15805484
21. Chickarmane VS, Gordon SP, Tarr PT, Heisler MG, Meyerowitz EM. Cytokinin signaling as a positional cue for patterning the apical-basal axis of the growing Arabidopsis shoot meristem. Proc Natl Acad Sci USA. 2012;109(10):4002–7.

PubMed PMID: 11060241
22. Takada S, Hibara K, Ishida T, Tasaka M. The CUP-SHAPED COTYLEDON1 gene of Arabidopsis regulates shoot apical meristem formation. Development. 2001;128(7):1127–35. PubMed PMID: 11245578
23. Vernoux T, Kronenberger J, Grandjean O, Laufs P, Traas J. PIN-FORMED 1 regulates cell fate at the periphery of the shoot apical meristem. Development. 2000;127(23):5157–65. PubMed PMID: 22345559
24. Yadav RK, Tavakkoli M, Xie M, Girke T, Reddy GV. A high-resolution gene expression map of the Arabidopsis shoot meristem stem cell niche. Development. 2014;141(13):2735-44. PubMed PMID: 24961803
25. Brewer PB. PETAL LOSS, a trihelix transcription factor gene, regulates perianth architecture in the Arabidopsis flower. Development. 2004;131:4035–45. PubMed PMID: 15269176
26. Abley K, Sauret-Güeto S, Marée AF, Coen E. Formation of polarity convergences underlying shoot outgrowths. Elife. 2016;5. PMID: 27478985
27. Matsumoto N, Okada K. A homeobox gene, PRESSED FLOWER, regulates lateral axis-dependent development of Arabidopsis flowers. Genes Dev. 2001;15(24):3355–64. PubMed PMID: 11751640
28. Caggiano MP, Yu X, Bhatia N, Larsson A, Ram H, Ohno CK, et al. Cell type boundaries organize plant development. eLife. 2017;6. PubMed PMID: 28895530
29. Siegfried KR, Eshed Y, Baum SF, Otsuga D, Drews GN, Bowman JL. Members of the YABBY gene family specify abaxial cell fate in Arabidopsis. Development. 1999;126(18):4117-28. PubMed PMID: 10457020
30. Sawa S, Watanabe K, Goto K, Kanaya E, Morita EH, Okada K. FILAMENTOUS FLOWER, a meristem and organ identity gene of Arabidopsis, encodes a protein with a zinc finger and HMG-related domains. Genes & Development. 1999;13:1079–88. PubMed PMID: 10323860
31. Smith HMS, Hake S. The interaction of two homeobox genes, BREVIPEDICELLUS and PENNYWISE, regulates internode patterning in the Arabidopsis inflorescence. Plant Cell. 2003;15(8):1717–27. PubMed PMID: 12897247
32. Zürcher E, Liu J, di Donato M, Geisler M, Müller B. Plant development regulated by cytokinin sinks. Science. 2016;353(6303):1027–30. PubMed PMID: 27701112
33. Abe M, Katsumata H, Komeda Y, Takahashi T. Regulation of shoot epidermal cell differentiation by a pair of homeodomain proteins in Arabidopsis. Development.

2003;130(4):635–43. PubMed PMID: 12505995

34. Nakamura M, Katsumata H, Abe M, Yabe N, Komeda Y, Yamamoto KT, et al. Characterization of the class IV homeodomain-Leucine Zipper gene family in Arabidopsis. Plant Physiol. 2006;141(4):1363–75. PubMed PMID: 16778018
35. Hay A, Kaur H, Phillips A, Hedden P, Hake S, Tsiantis M. The gibberellin pathway mediates KNOTTED1-type homeobox function in plants with different body plans. Curr Biol. 2002;12(18):1557–65. PubMed PMID: 12372247
36. Saddic LA, Huvermann B, Bezhani S, Su Y, Winter CM, Kwon CS, et al. The LEAFY target LMI1 is a meristem identity regulator and acts together with LEAFY to regulate expression of CAULIFLOWER. Development. 2006;133(9):1673–82. PubMed PMID: 16554366
37. Zhao Y, Medrano L, Ohashi K, Fletcher JC, Yu H, Sakai H, et al. HANABA TARANU is a GATA transcription factor that regulates shoot apical meristem and flower development in Arabidopsis. Plant Cell. 2004;16(10):2586–600. PubMed PMID: 15367721
